# Supplementary material for: CDK8/19 inhibition prevents adaptive resistance to CDK4/6 inhibitors in vitro and in vivo
Source: Cell Rep Med. 2026 Aug 19;7(9):103004. doi: 10.1016/j.xcrm.2026.103004 (PMC13589481; doi:10.1016/j.xcrm.2026.103004)
Supplement: Supplementary file 1 [file mmc1.pdf]

Cell Reports Medicine, Volume *7*

Supplemental information

CDK8/19 inhibition prevents adaptive resistance to CDK4/6 inhibitors *in vitro* and *in vivo*

Zachary T. Mack, Margarita Yastrebova, Chang-uk Lim, Xiaokai Ding, Jing Li, Alvina Khamidullina, Amanda C. Sharko, Michael Saint-Antoine, Thomas A. Hilimire, Gary P. Schools, Kaitlyn Digsby, Amanda Eckstrom, Erin Belcher, Gwenael Madiot, Hao Ji, Vitali Sikirzhitski, Chao Cai, Guoshuai Cai, Abhyudai Singh, Victor Tatarskiy, Mengqian Chen, Igor B. Roninson, and Eugenia V. Broude

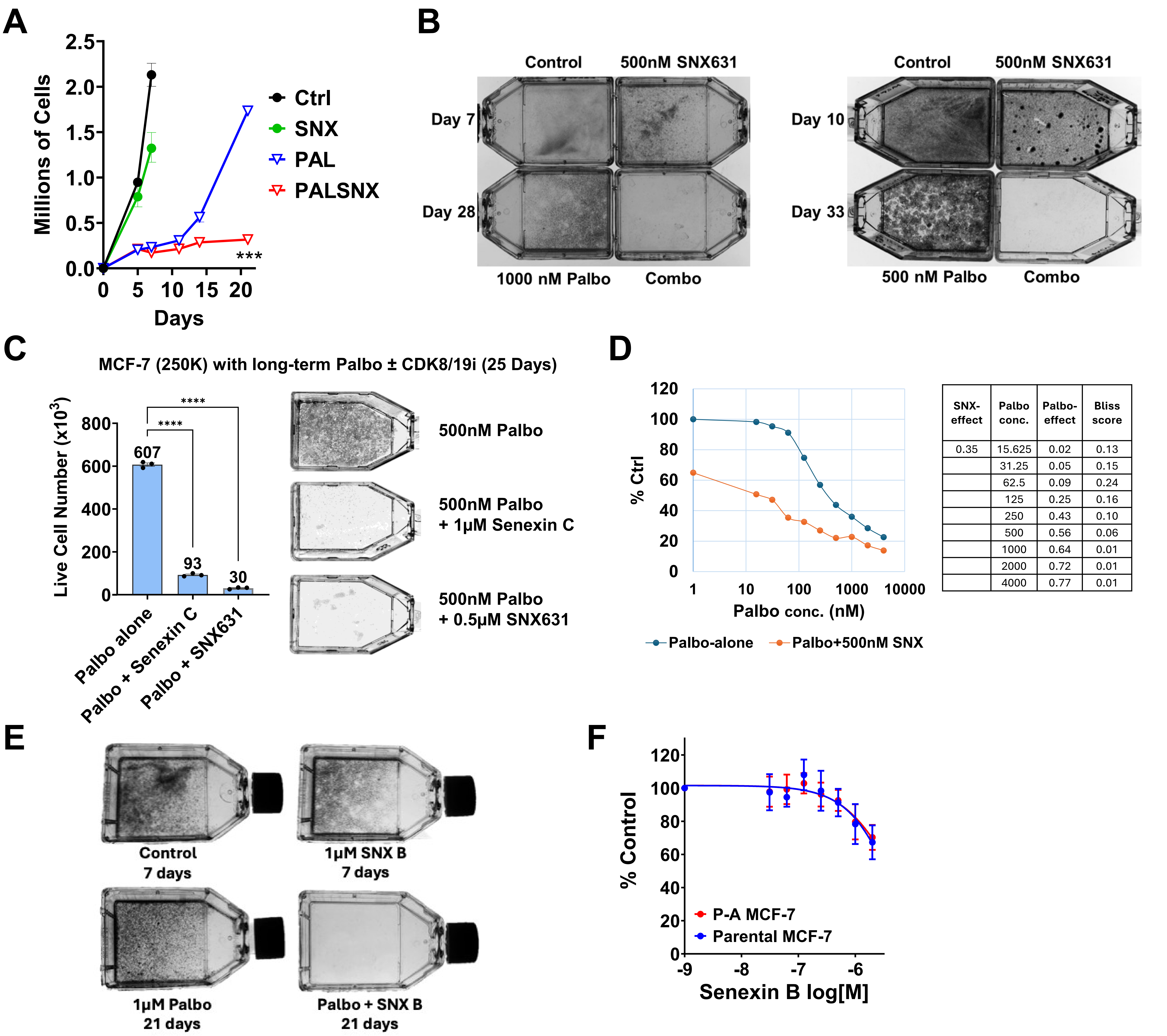

Figure S1. CDK8/19 inhibitors enhance the long-term growth suppression of palbociclib in MCF-7 cells. Related to Figures 1. **(A)** Raw cell counts from 20-day viability analyses of MCF-7 cells treated with 500 nM palbociclib with or without 500 nM SNX631. Statistics: unpaired two-tailed Welch's t-test; ***: $P < 0.001$. **(B)** Long-term palbociclib-treated (500 or 1000 nM) and combination-treated flasks were stained with crystal violet once apparent palbociclib adaptation was observed in the palbociclib-treated flasks. **(C)** Final cell counts and crystal violet-stained flasks after 25 days of treatment of MCF-7 cells with 500 nM palbociclib alone or with 1 µM Senexin C or 500 nM SNX631. Statistics: ordinary one-way ANOVA with Šídák's multiple comparisons test; ****: $P < 0.0001$. **(D)** Synergy analysis of different concentrations of palbociclib combined with 500 nM SNX631 in MCF-7 cells. **(E)** Representative images of crystal violet-stained flasks. Untreated and 1 µM Senexin B (SNX B)-treated MCF-7 cells were stained at 7 days, while cells treated with 1 µM palbociclib with and without Senexin B were stained at 21 days. **(F)** Seven-day growth assay of parental and palbociclib-adapted (P-A) MCF-7 cells treated with Senexin B at different concentrations. Data are represented as mean +/- SEM.

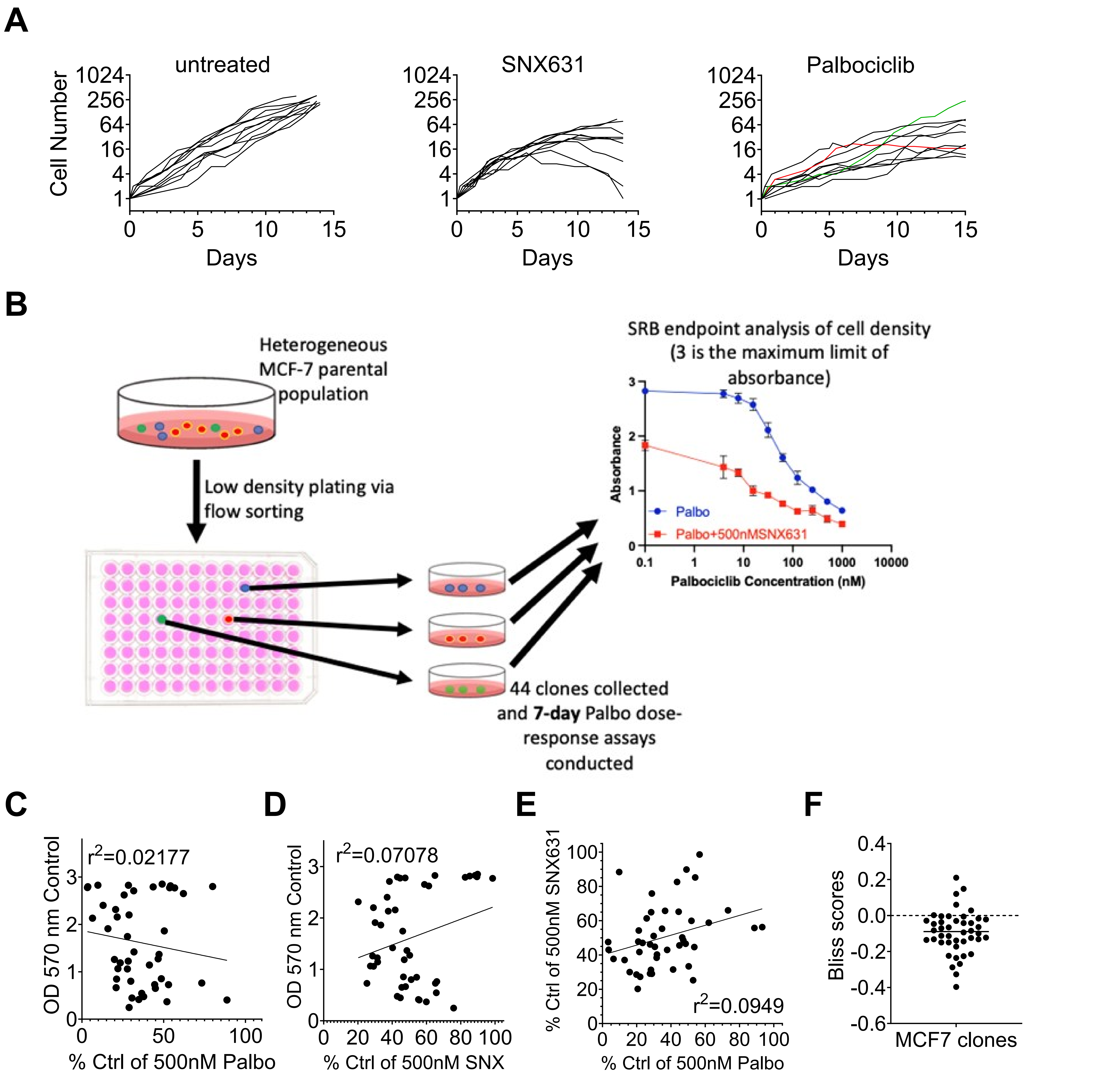

Figure S2. Clonal heterogeneity in MCF-7 cell responses to palbociclib and CDK8/19 inhibition. Related to Figures 2.

(A) Real-time video analysis of 10 clusters of untreated cells (left), cells treated with 500 nM SNX631 (middle), or 500 nM palbociclib (right). Each line represents a cluster originating from a single cell, with green and red lines highlighting clusters of interest under palbociclib treatment. **(B)** Diagram of subcloning of MCF-7 cells; 44 subpopulations were generated using low-density plating. **(C–D)** Correlation curves visualizing characteristics of MCF-7 clones following a 7-day SRB viability assay. Correlation coefficients were determined by linear regression analysis in GraphPad Prism 10. **(E)** Correlation of the effects of 500 nM palbociclib and 500 nM SNX631 on 44 MCF-7 clones (7-day cell growth assay). **(F)** Synergy analysis of 44 MCF-7 clones showing Bliss scores calculated from the effects of 500 nM palbociclib and 500 nM SNX631. Data are represented as mean +/- SEM.

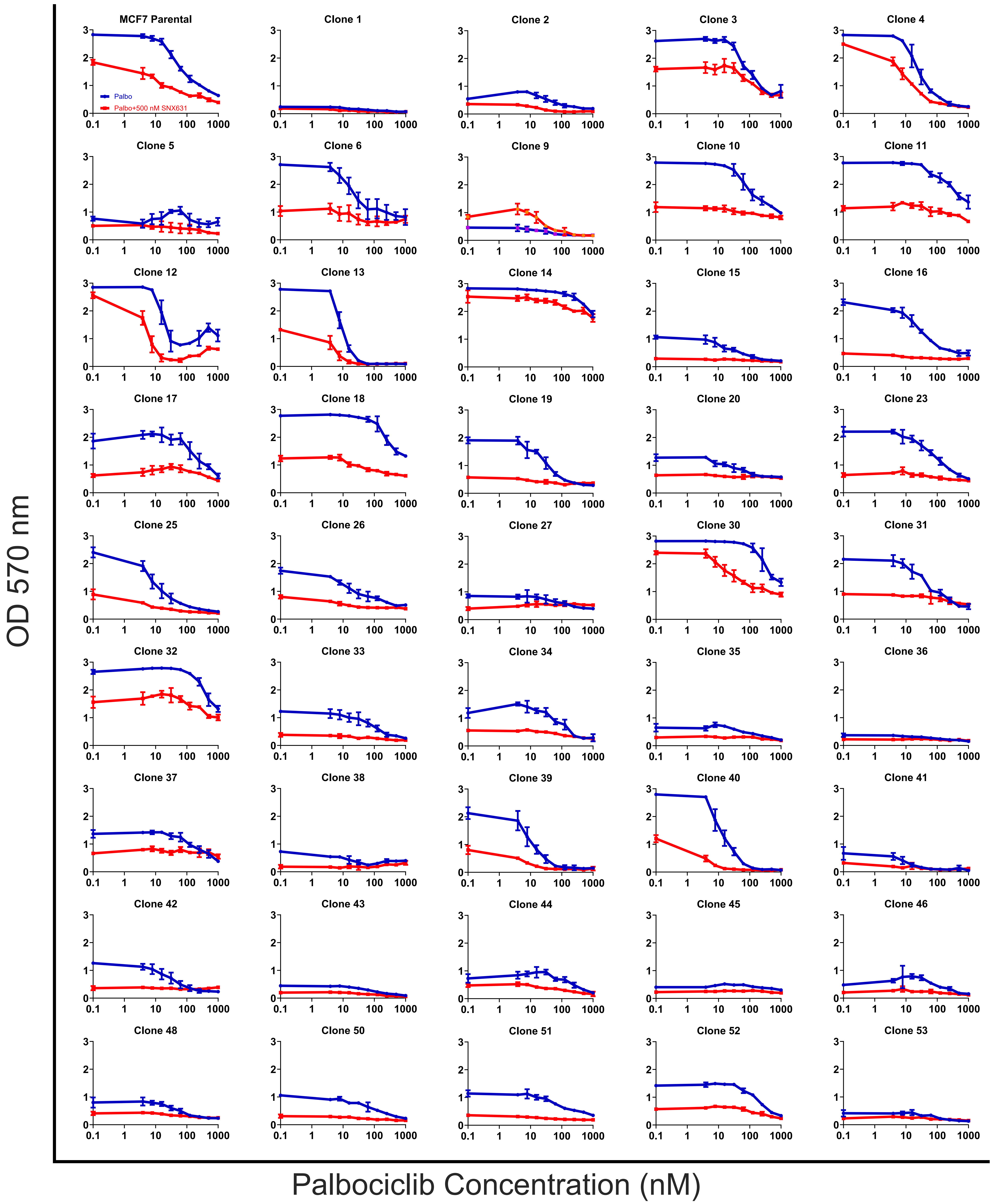

Figure S3. Drug response profiling of single-cell MCF-7 clones to palbociclib and CDK8/19 inhibition. Related to Figures 2.

Single-cell clones were isolated and tested for their sensitivity to palbociclib, SNX631, and the combination, and compared to their parental population (top left). Parameters of the dose–response curves were determined using the IC50 of the parental population. Data are represented as mean +/- SEM.

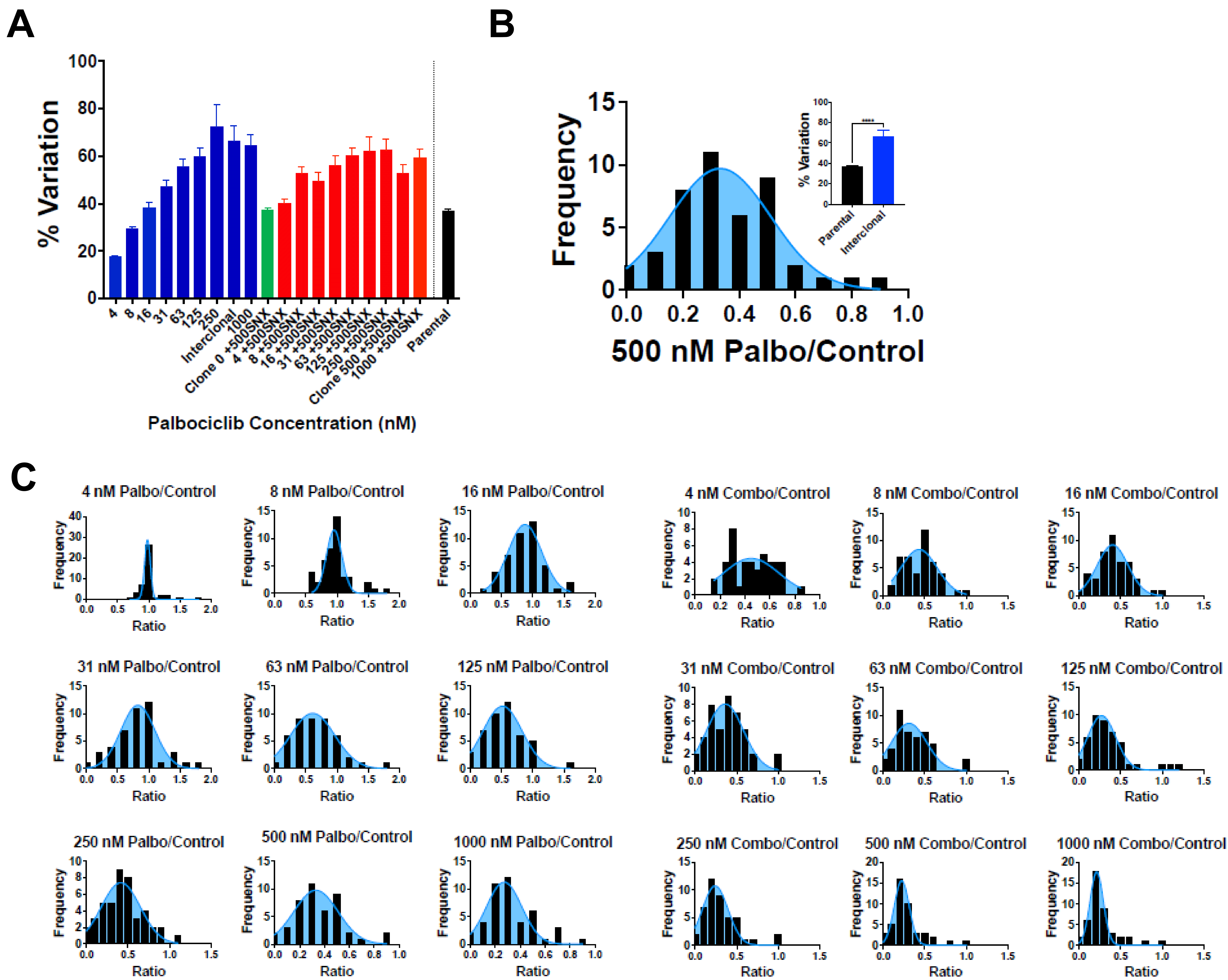

Figure S4. Adaptation to palbociclib occurs regardless of transient sensitivity. Related to Figures 2.

(A) A Percent coefficient of variation between the 44 clones' response to each palbociclib and combination treatment group compared to variation between 10 biological repeats of MCF-7 parental cell line to 500 nM palbociclib. **(B)** Histogram displaying the distribution of relative drug sensitivity to 500 nM palbociclib across 44 MCF-7 subclones. A Gaussian distribution was fit to the data using GraphPad Prism's nonlinear regression function. **(C)** Histograms displaying the distribution of relative drug sensitivity at various concentrations of palbociclib and SNX631+palbociclib combination. A Gaussian distribution was fit to the data using GraphPad Prism's nonlinear regression function. Data are represented as mean +/- SEM.

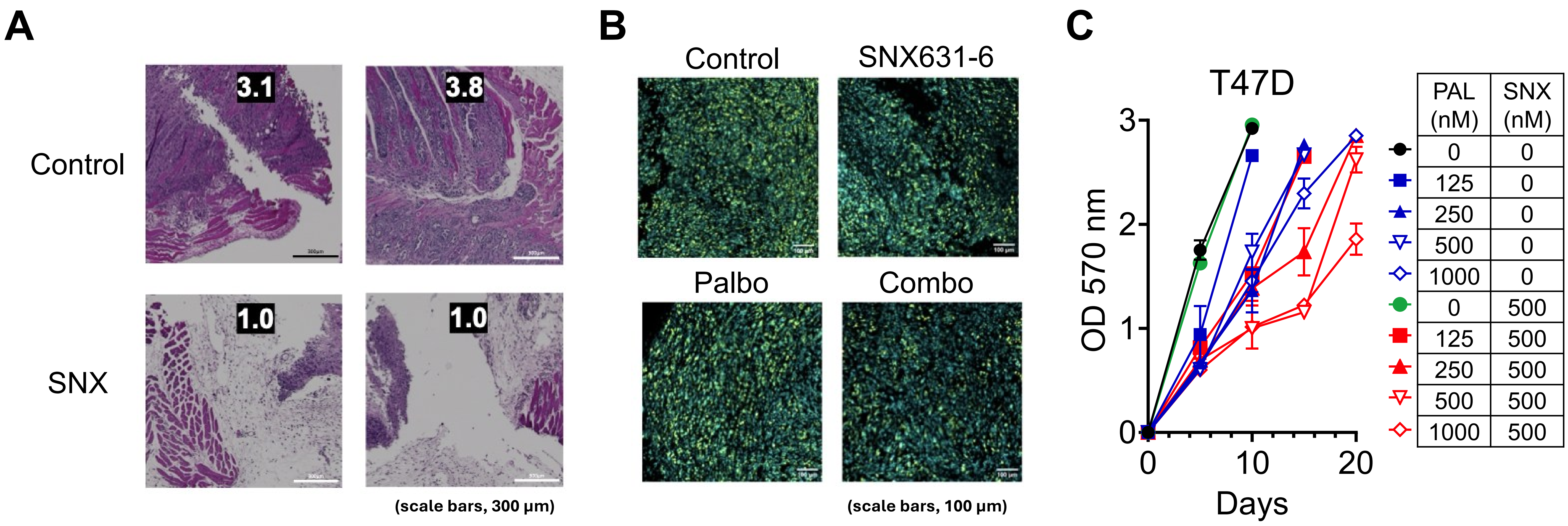

Figure S5. Effects of CDK8/19 inhibition on MCF-7 tumor invasion in vivo and on palbociclib responses in T47D cell proliferation *in vitro*. Related to Figures 3.

(A) Histological sections of dissected tumors stained with hematoxylin and eosin showing representative images and corresponding invasion scores (top left of each image) for MCF-7 tumors invading abdominal muscle in control (top) versus SNX631-6-treated (bottom) mice. Invasion scores, ranging from 0 (no invasion) to 5 (extensive invasion), were assigned based on the depth of tumor infiltration into muscle tissue (n = 10 tumors per treatment group). **(B)** Representative composite images of Ki67 (yellow)-labeled sections counterstained with DAPI (blue). **(C)** Twenty-day Sulforhodamine B (SRB) viability analyses performed on T47D cells treated with concentrations greater than their IC50 of palbociclib with (red lines) and without (blue lines) 500 nM SNX631. Data are represented as mean +/- SEM.

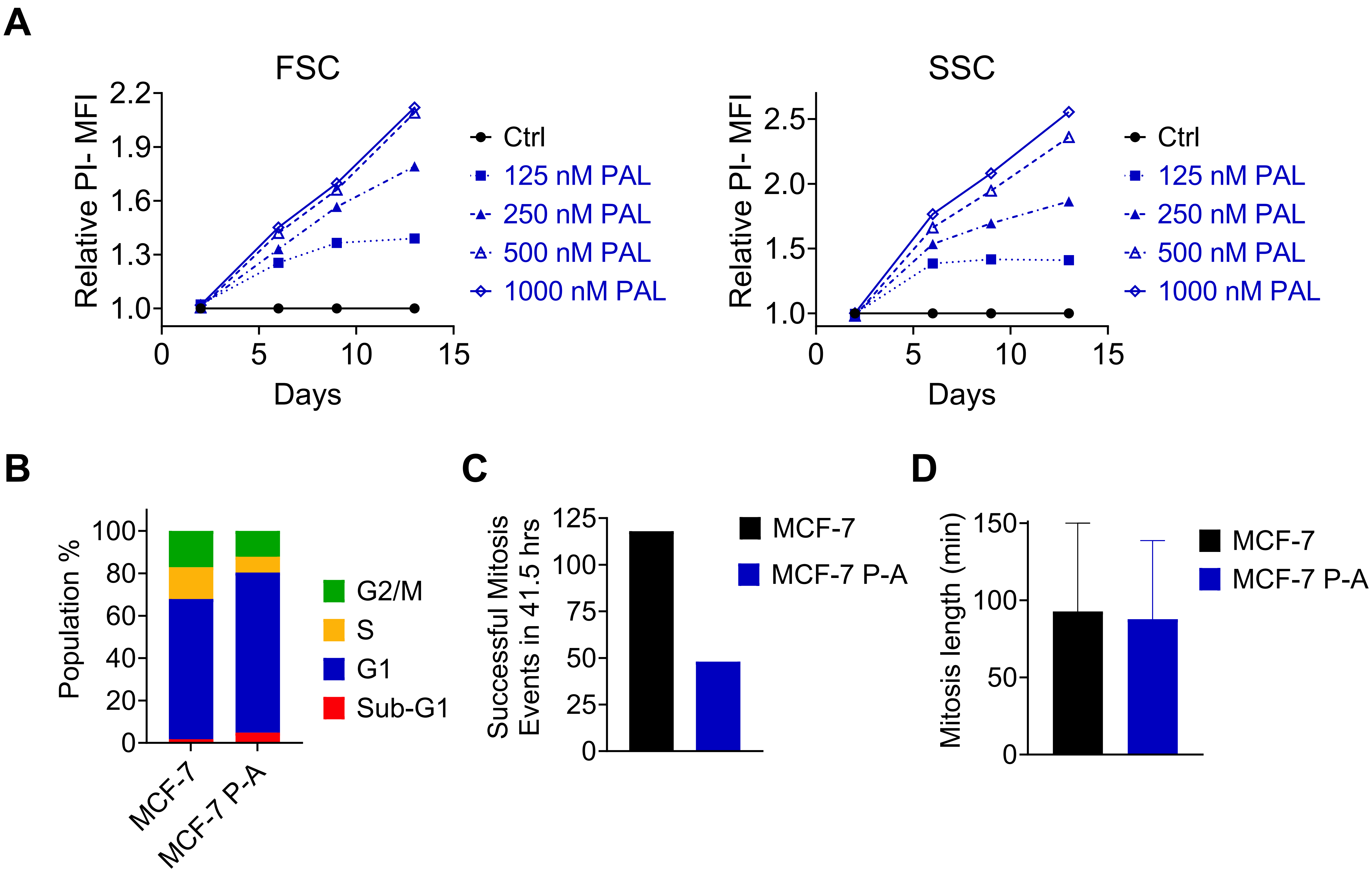

Figure S6. Effects of long-term palbociclib treatment on cell size, cell-cycle distribution, and mitotic dynamics in MCF-7 cells. Related to Figures 4.

(A) Forward scatter (FSC; left) and side scatter (SSC; right) quantification of palbociclib's effect on cell size and internal complexity over time by flow cytometric analysis, measured as mean fluorescence intensity (MFI). **(B)** Cell-cycle profile of MCF-7 cells treated with 0.1% DMSO vehicle control or 500 nM palbociclib for 42 days. **(C–D)** Number (**C**) and duration (**D**) of mitosis events quantified by a 41.5-hour microscope image time course of control and 42-day palbociclib-adapted (P-A) groups.

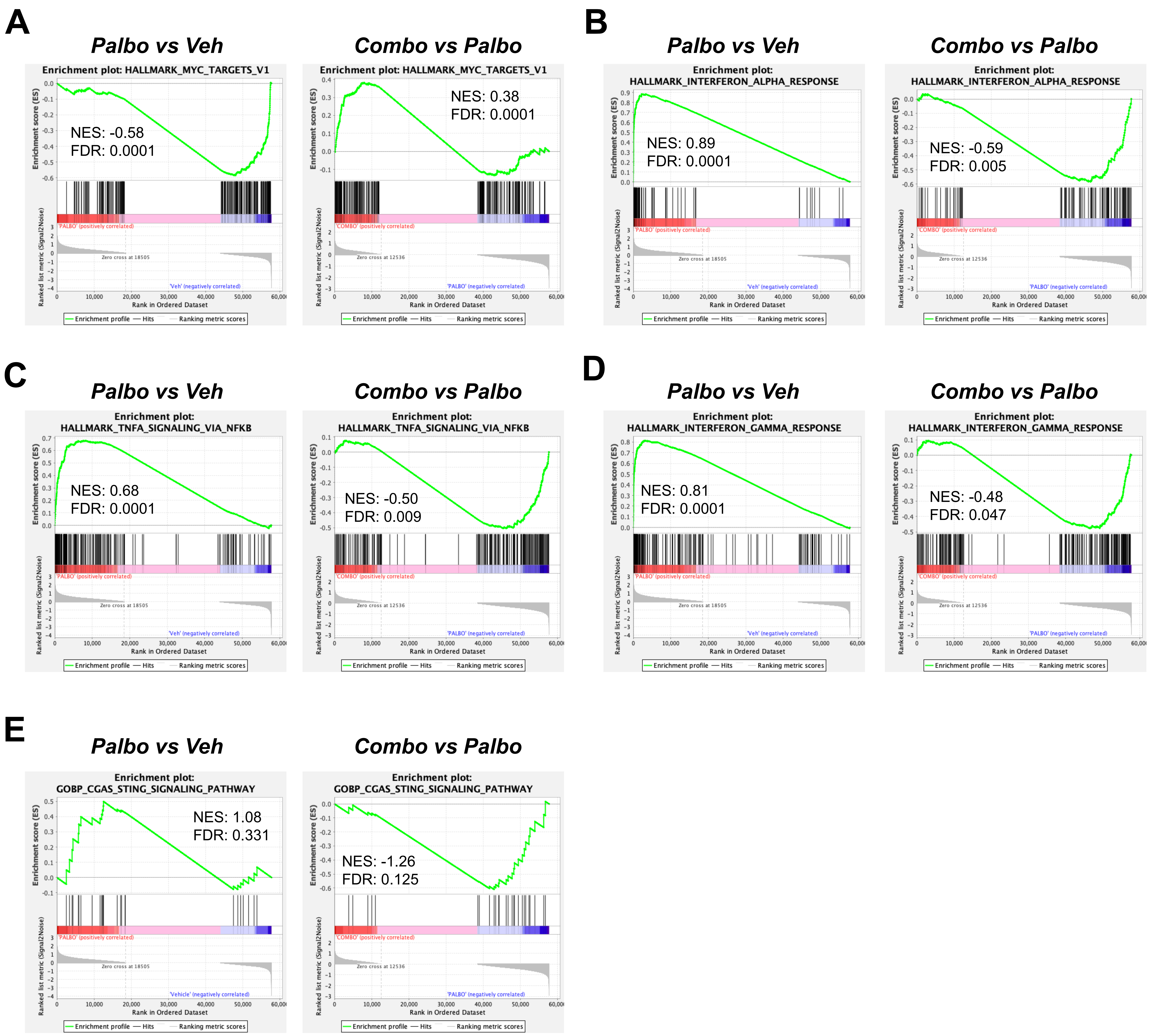

Figure S7. Gene set enrichment analysis (GSEA) of signaling pathways in MCF-7 tumors treated with palbociclib or combination therapy *in vivo*. Related to Figures 6.

(A–E) Enrichment score plots of GSEA Hallmark gene sets for MYC Targets V1 (**A**), Interferon-alpha (**B**), Interferon-gamma (**C**), TNF-alpha (**D**), and cGAS–STING (**E**) pathways comparing *in vivo* palbociclib vs vehicle (left) and *in vivo* combination vs palbociclib (right).

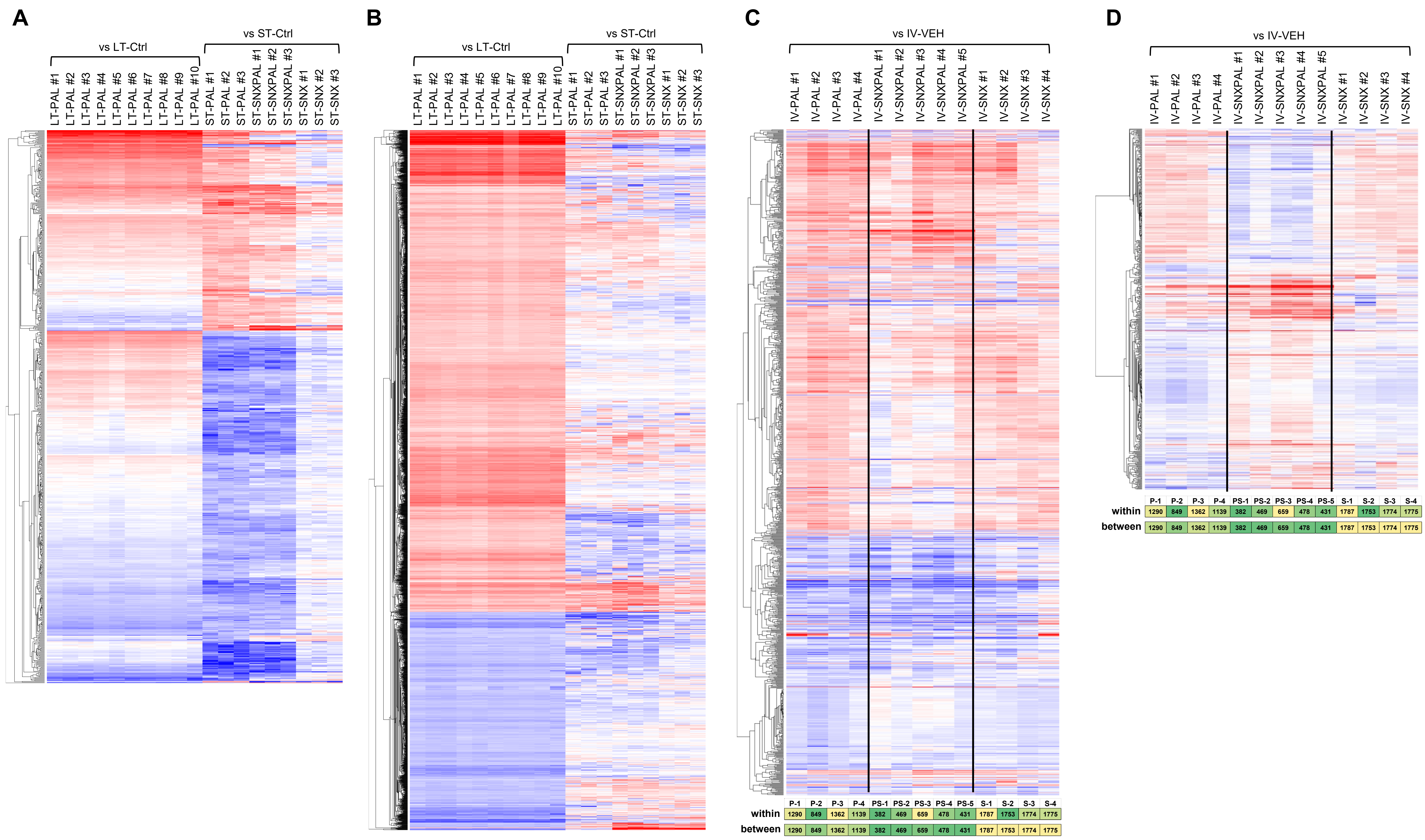

Figure S8. Transcriptomic profiling of palbociclib treatment and its combination with CDK8/19i *in vitro* and *in vivo*. Related to Figure 6

(A–D) Heatmaps displaying individual replicate fold change relative to corresponding control samples with unsupervised hierarchical clustering of 711 DEGs for ST-PAL vs ST-DMSO (**A**), 3,599 DEGs for LT-PAL vs LT-DMSO (**B**), 1,023 DEGs for IV-Palbo vs IV-Vehicle (**C**), and 515 DEGs for IV-Combo vs IV-Palbo (**D**). IV plots include terminal tumor sizes listed above each corresponding replicate and conditionally formatted to label the largest (yellow) to smallest (green) tumors within (top) and between (bottom) treatment groups. DEGs were defined by a fold change >1.5 and an FDR <0.05. Heatmaps were generated using Morpheus (https://software.broadinstitute.org/morpheus).

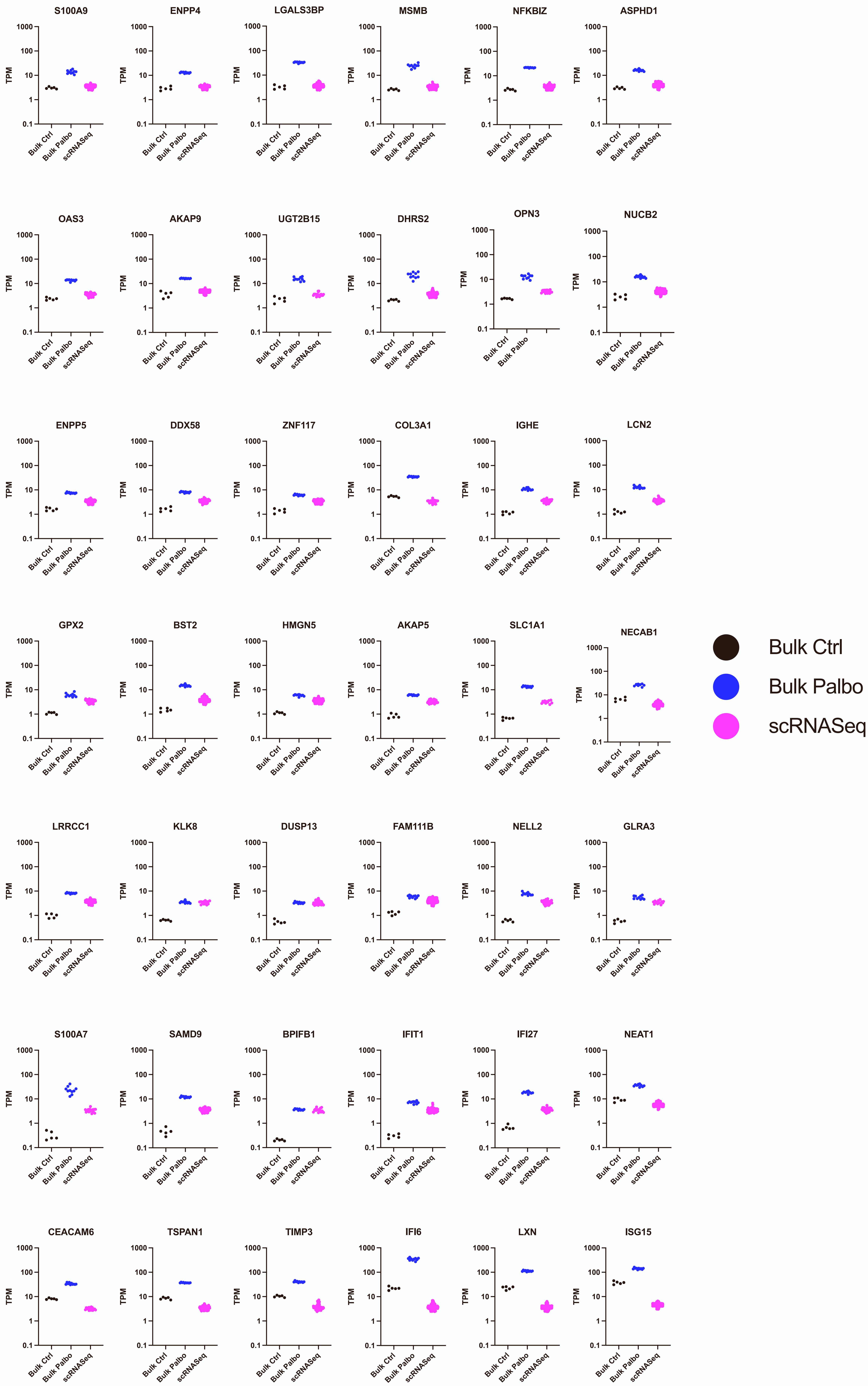
S100A9
ENPP4
LGALS3BP
MSMB
NFKBIZ
ASPHD1
OAS3
AKAP9
UGT2B15
DHRS2
OPN3
NUCB2
ENPP5
DDX58
ZNF117
COL3A1
IGHE
LCN2
GPX2
BST2
HMGN5
AKAP5
SLC1A1
NECAB1
LRRCC1
KLK8
DUSP13
FAM111B
NELL2
GLRA3
S100A7
SAMD9
BPIFB1
IFIT1
IFI27
NEAT1
CEACAM6
TSPAN1
TIMP3
IFI6
LXN
ISG15
TPM
Bulk Ctrl
Bulk Palbo
scRNASeq

Figure S9. Single-cell expression patterns of long-term palbociclib–associated DEGs in MCF-7 cells. Related to Figure 6

(A) Dot plots of 42 DEGs identified in LT-PAL (log2(FC) ≥ 2; FPKM > 5) with nonzero expression in 977 single MCF-7 cells, generated using Prism 10. Plots are ordered from top left to bottom right according to the similarity of single-cell TPM expression to bulk LT-DMSO RNA-seq profiles.
