# Supplementary material for: CDK8/19 inhibition prevents adaptive resistance to CDK4/6 inhibitors in vitro and in vivo
Source: Cell Rep Med. 2026 Aug 19;7(9):103004. doi: 10.1016/j.xcrm.2026.103004 (PMC13589481; doi:10.1016/j.xcrm.2026.103004)
Supplement: Supplementary file 2 [file mmc6.pdf]

Cell Reports Medicine

Article

CDK8/19 inhibition prevents adaptive resistance to CDK4/6 inhibitors *in vitro* and *in vivo*

Graphical abstract

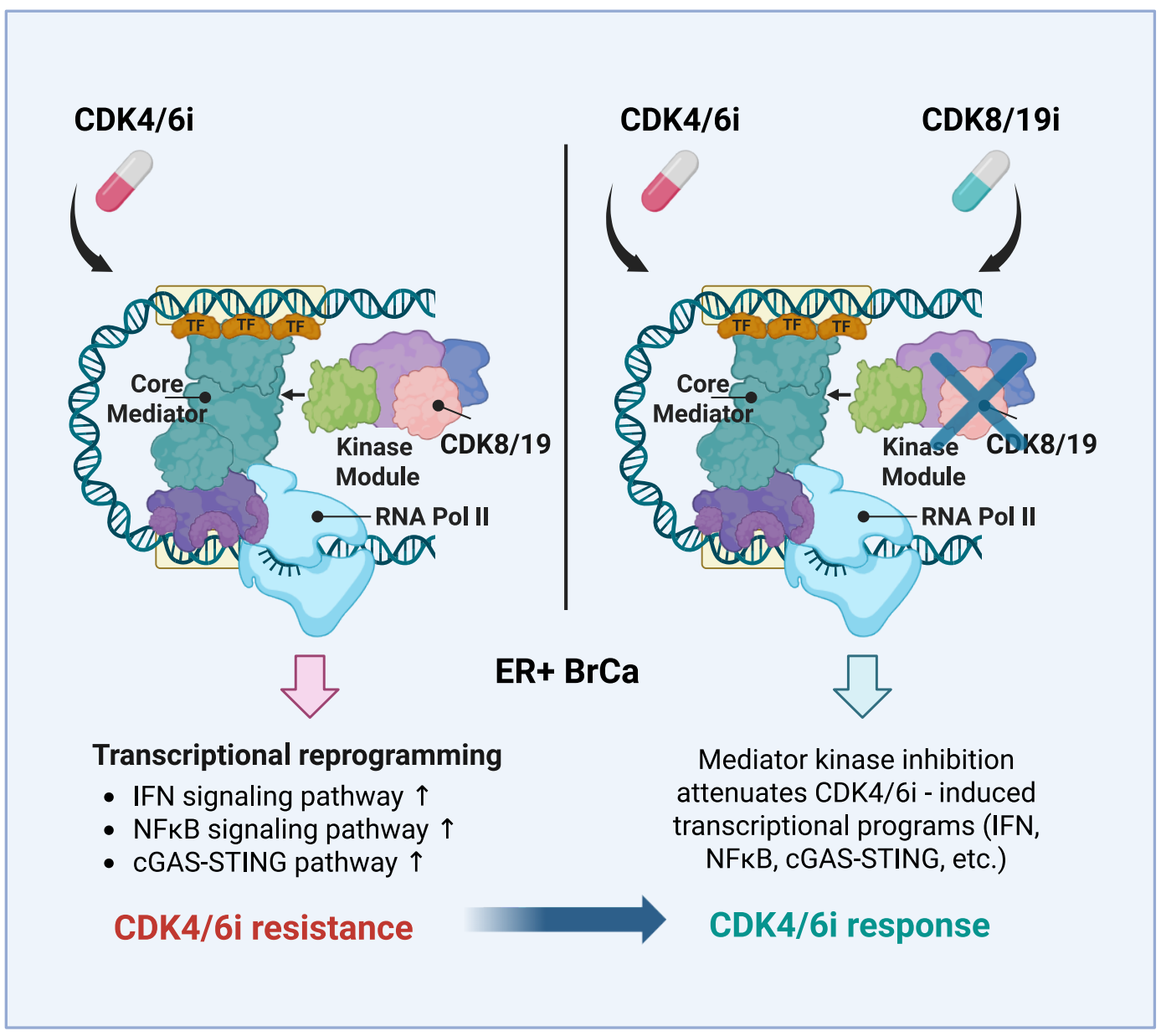

Authors

Zachary T. Mack, Margarita Yastrebova, Chang-uk Lim, ..., Mengqian Chen, Igor B. Roninson, Eugenia V. Broude

Correspondence

chenm@cop.sc.edu (M.C.),
roninsoni@cop.sc.edu (I.B.R.),
broude@cop.sc.edu (E.V.B.)

In brief

Non-genetic adaptation curtails CDK4/6 inhibitor therapy. Mack et al. show that adaptive resistance arises through transcriptional reprogramming and is suppressed by inhibition of the Mediator kinases CDK8/19, which enhances CDK4/6 inhibitor efficacy *in vitro* and *in vivo*.

Highlights

- CDK4/6 inhibitor resistance rapidly emerges through non-genetic adaptation
- Resistance involves reversible transcriptional state switching
- CDK8/19 inhibition suppresses adaptation-associated transcriptional reprogramming
- CDK8/19 inhibitors enhance and prolong CDK4/6 inhibitor efficacy *in vivo*

Mack et al., 2026, Cell Reports Medicine *7*, 103004
September 15, 2026 © 2026 The Author(s). Published by Elsevier Inc.
https://doi.org/10.1016/j.xcrm.2026.103004

CellPress

Cell Reports Medicine

Cell Press
OPEN ACCESS

Article

CDK8/19 inhibition prevents adaptive resistance to CDK4/6 inhibitors *in vitro* and *in vivo*

Zachary T. Mack,[1] Margarita Yastrebova,[2] Chang-uk Lim,[1] Xiaokai Ding,[1,8] Jing Li,[1,9] Alvina Khamidullina,[2] Amanda C. Sharko,[1] Michael Saint-Antoine,[3,10] Thomas A. Hilimire,[1,4] Gary P. Schools,[1] Kaitlyn Digsby,[1,11] Amanda Eckstrom,[1,12] Erin Belcher,[1] Gwenael Madiot,[1] Hao Ji,[1] Vitali Sikirzhitski,[1] Chao Cai,[5] Guoshuai Cai,[6,7] Abhyudai Singh,[3] Victor Tatarskiy,[2] Mengqian Chen,[1,4,13,*] Igor B. Roninson,[1,*] and Eugenia V. Broude[1,*]

[1]Department of Drug Discovery and Biomedical Sciences, College of Pharmacy, University of South Carolina, Columbia, SC, USA
[2]Institute of Gene Biology, Russian Academy of Sciences, Moscow, Moscow, Russian Federation
[3]Department of Electrical and Computer Engineering, Department of Biomedical Engineering, College of Engineering, University of Delaware, Newark, DE, USA
[4]Senex Biotechnology, Inc., Columbia, SC, USA
[5]Department of Clinical Pharmacy and Outcomes Sciences, College of Pharmacy, University of South Carolina, Columbia, SC, USA
[6]Department of Surgery, College of Medicine, University of Florida, Gainesville, FL, USA
[7]Department of Biostatistics, College of Public Health and Health Professions, University of Florida, Gainesville, FL, USA
[8]Present address: The Gene Lay Institute of Immunology and Inflammation, Brigham and Women's Hospital, Massachusetts General Hospital and Harvard Medical School, Boston, MA, USA
[9]Present address: Center for Translational Pathology, University of Michigan, Ann Arbor, Michigan, USA
[10]Present address: Chronobiology and Sleep Institute, University of Pennsylvania, Philadelphia, PA 19104, USA
[11]Present address: Department of Pharmacology, University of Michigan, Ann Arbor, Michigan, USA
[12]Present address: Department of Leukemia, Division of Cancer Medicine, The University of Texas MD Anderson Cancer Center, Houston, Texas, USA
[13]Lead contact
*Correspondence: chenm@cop.sc.edu (M.C.), roninsoni@cop.sc.edu (I.B.R.), broude@cop.sc.edu (E.V.B.)
https://doi.org/10.1016/j.xcrm.2026.103004

SUMMARY

Cyclin-dependent kinase 4/6 (CDK4/6) inhibitors have become a standard of care for estrogen receptor-positive breast cancer and are being developed for other malignancies. However, resistance to these drugs readily develops, limiting their impact on patient survival. Mechanisms of resistance to CDK4/6 inhibition involve multiple changes in gene expression. We investigated the process of tumor cell adaptation to CDK4/6 inhibitors and the impact of selective inhibitors of CDK8/19 Mediator kinases—broad-spectrum regulators of transcriptional reprogramming—on such adaptations. Adaptive non-genetic resistance to CDK4/6 inhibitors develops rapidly, but the addition of CDK8/19 inhibitors prevents the development of this resistance in different tumor models, *in vitro* and *in vivo*. RNA sequencing (RNA-seq) analysis reveals that combining CDK4/6 and CDK8/19 inhibitors suppresses many of the adaptation-associated changes in gene expression, including those previously associated with CDK4/6 inhibitor resistance. The findings suggest that CDK8/19 inhibition may greatly extend the therapeutic benefit of CDK4/6 inhibitors.

INTRODUCTION

Estrogen receptor-positive breast cancers (ER^+ BrCa) make up 70% of all breast cancers in the US. The most recent major class of drugs approved for ER^+ BrCa patients target cyclin-dependent kinases (CDKs) 4 and 6, which mediate G1/S checkpoint transition. CDK4/6 inhibitors (CDK4/6i) arrest the cell cycle and induce senescence by inhibiting phosphorylation of the Rb protein by the CDK4/6-cyclin D complex.[1] Three CDK4/6i—palbociclib, ribociclib, and abemaciclib—have been approved for the treatment of ER^+ BrCa. Clinical and laboratory studies have shown that long-term treatment with CDK4/6i,[2–4] as well as with a recently developed CDK4-specific inhibitor, atirmociclib,[5] leads to drug resistance and reduced drug efficacy. It is now understood that the resistance to targeted drugs may first emerge as a non-genetic adaptation of tumor cells before stable resistant mutants can be selected.[6–8] Early adaptation to CDK4/6 inhibition, evidenced by continued S-phase entry in treated ER^+ BrCa, can occur as early as 72 h.[9]

Mechanisms of both stable genetic resistance and transient transcriptional adaptation to CDK4/6i are varied and include acquired CDK6 amplification or overexpression,[10–13] hyperphosphorylation of Rb by CDK2-cyclin E,[14] gradual loss of Rb protein,[15] and amplification of PI3K/mTOR signaling[16,17] and E2F activity[18]; such gradual transcriptional changes can be suppressed through pleiotropic transcriptional inhibition, in

© 2026 The Author(s). Published by Elsevier Inc.
This is an open access article under the CC BY license (http://creativecommons.org/licenses/by/4.0/).

particular via inhibitors of CDK7[19,20] and CDK9.[21] Palbociclib resistance has also been linked to the development of the senescence-associated secretory phenotype (SASP), whereby senescent cells secrete factors enabling the survival of their neighbors.[22] Given the diversity of the mechanisms for CDK4/6i resistance, blocking any individual resistance mechanisms is unlikely to have a major impact on patient outcomes. Thus, a combinatorial treatment regimen that would prevent the multiple mechanisms of resistance to CDK4/6i simultaneously could greatly improve the current standard of care.

CDK8 or its paralog, CDK19, together with their binding partner, cyclin C (CCNC), and the proteins MED12 and MED13, form the regulatory CDK module of the transcriptional Mediator complex.[23] CDK8 and CDK19 Mediator kinases differ in their expression but have qualitatively similar functions, and both paralogs need to be inhibited to achieve a phenotypic effect.[24] In contrast to CDK4/6, CDK2, or CDK1, CDK8 and CDK19 regulate transcription but do not directly regulate cell cycle progression.[23] Unlike other transcriptional CDKs, such as CDK7 or CDK9, Mediator kinases are not a part of the overall transcription machinery but regulate the Mediator complex levels and act as cofactors or modifiers of several transcription factors. In particular, CDK8 and CDK19 act as positive regulators of signal-induced transcription factors, such as NF-κB, STATs, HIF1A, and ER, by promoting C-terminal domain phosphorylation of RNA Pol II, enabling pause-release and the elongation of transcription selectively in the context of genes activated by these transcription factors.[24–28] Through this mechanism, CDK8 and CDK19 were also shown to mediate SASP.[29] Importantly, CDK8/19 inhibition downregulates primarily the signal-induced, but not basal, transcription of signal-activated genes, leading to the identification of CDK8/19 as pleiotropic regulators of transcriptional reprogramming.[24,25] CDK8 and CDK19 also act as negative regulators of transcription, through post-transcriptional regulation of the Mediator complex subunits.[24]

Consistent with the effect of Mediator kinases on transcriptional reprogramming, CDK8/19 inhibitors (CDK8/19i), a new class of agents undergoing clinical trials in solid tumors and leukemias (clinicaltrials.gov: NCT03065010, NCT04021368, NCT05052255, and NCT05300438), were found to prevent the development of resistance, and sometimes help overcome the already acquired resistance, to different classes of anticancer agents, including, among others, antiestrogens, androgen deprivation, ionizing radiation, and inhibitors of HER2, EGFR, MEK, ERK/MAPK, mTOR, and AKT.[26,30–36] In light of these findings, we hypothesized that CDK8/19 inhibition could also prevent or help overcome the multiple non-genetic mechanisms implicated in tumor resistance to CDK4/6i. In the present study, we investigated the process of rapid non-genetic adaptation of tumor cells to CDK4/6i and the effect of CDK8/19i on such adaptations. Our results show that the non-genetic adaptation of tumor cells to palbociclib and other CDK4/6i is a robust, non-stochastic process driven by transcriptional reprogramming, which is fully prevented by the addition of CDK8/19i, both *in vitro* and *in vivo*. These results warrant the exploration of CDK8/19i administration as a general approach to preventing the development of resistance and extending the therapeutic efficacy of CDK4/6i.

RESULTS

Tumor cells rapidly adapt to CDK4/6 inhibitors, and this adaptation is prevented by Mediator kinase inhibition

To investigate tumor cell adaptation to CDK4/6i, the ER^+ BrCa cell lines MCF-7 and ZR-75-1 and the colorectal cancer cell line SW-620 were chosen for their *de novo* sensitivity to CDK4/6i palbociclib.[37] To inhibit CDK8/19 Mediator kinase, the majority of our studies used equipotent selective CDK8/19i analogs SNX631[33] and SNX631-6,[36] with the latter being a late-preclinical stage drug candidate. Traditionally, palbociclib-resistant cell lines are selected for 4–12 months, usually with gradually increasing palbociclib concentrations.[10] However, we found that 20-day exposure to 125–500 nM palbociclib was sufficient to produce a loss of sensitivity in all three cell lines tested, as indicated by different assays. Strikingly, the addition of SNX631 at a concentration that produces only a weak growth inhibition by itself (500 nM) maintains growth arrest by all the tested palbociclib concentrations across all three cell lines (Figures 1A and 1B). The key results of these assays were confirmed by direct cell counting and crystal violet staining (Figures S1A–S2C). In 7-day assays, SNX631 produced only weak synergistic or additive effects with palbociclib (Figure S1D); its effects became highly significant only by day 19 (Figure 1A; Table S1), indicating the prevention of adaptive resistance rather than acute drug synergy.

Similar rapid adaptation and its prevention by SNX631 were observed with the other two FDA-approved CDK4/6i, ribociclib and abemaciclib (Figure 1C). The role of CDK8/19 inhibition in the prevention of palbociclib adaptation was confirmed using four other structurally distinct CDK8/19i, senexin B[26] (Figure S1E), senexin C,[38] BI1347,[39] and compound 25[40] (Figure 1D). These inhibitors, all of which act primarily on CDK8/19 but have different off-target effects, prevented palbociclib adaptation in MCF-7 cells, indicating that CDK819 was their common target responsible for the prevention of resistance.

Despite near-complete growth suppression by 500 nM palbociclib in MCF-7 cells after 7 days of treatment, drug-adapted colonies began to emerge by day 20, and the adapted cells reached confluence by day 42 (Figure 1E). Combination treatment with palbociclib and a CDK8/19i effectively prevented this adaptation. Notably, addition of SNX631 even 7 days after initiating palbociclib treatment markedly suppressed cell growth (Figure 1E), suggesting that Mediator kinase inhibition can either reverse early adaptive resistance or prevent its full establishment.

When MCF-7 cells adapted to 500 nM palbociclib for 42 days were replated into the same 20-day viability assay used for unselected cells, the resistance of these palbociclib-adapted (P-A) cells was not maintained (Figures 1F and 1G), indicating that the adaptive response was unstable and likely non-genetic, rather than a stable, mutation-driven resistance. The replated P-A cells underwent a similar adaptation process as unselected cells (Figure 1A), albeit at a slightly faster rate, and this adaptation was similarly prevented by co-treatment with 500 nM SNX631 (Figure 1G). The P-A cells retained the same sensitivity to CDK8/19i as the parental cells (Figure S1F).

CellPress
OPEN ACCESS

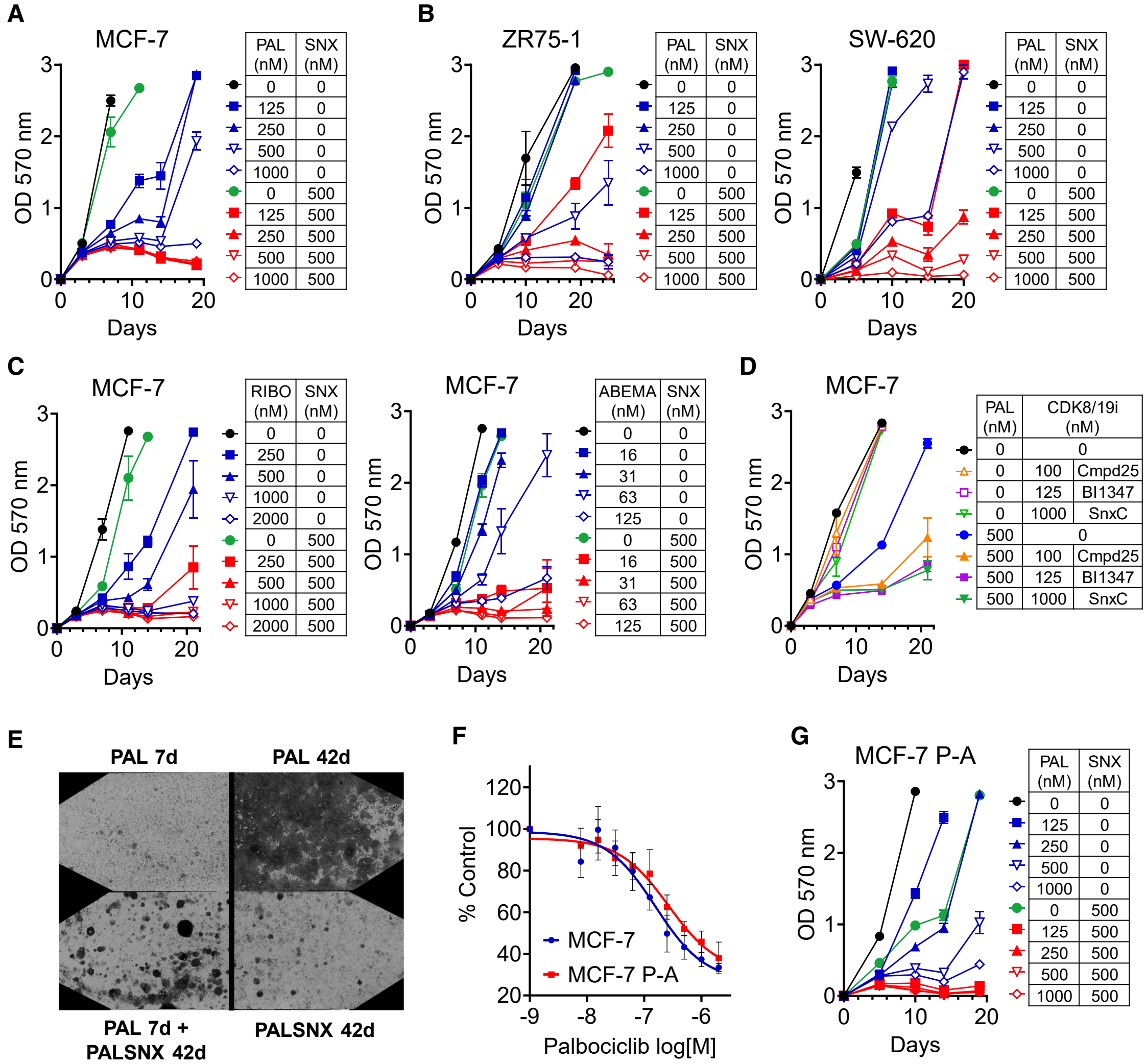

Figure 1. Palbociclib adaptation occurs rapidly and is prevented by CDK8/19 inhibition

(A and B) 20-day sulforhodamine B (SRB) viability assays of MCF-7(A), ZR-75-1, and SW-620 (B) cells treated with palbociclib with (red) or without (blue) 500 nM SNX631. 500 nM SNX631 monotherapy is in green. Results are representative of three independent experiments.

(C) 20-day SRB assays of MCF-7 cells treated with ribociclib (left) or abemaciclib (right).

(D) 21-day SRB assays of MCF-7 cells treated with the indicated CDK8/19 inhibitors ± palbociclib (PAL).

(E) Crystal violet-stained flasks of MCF-7 cells treated for 42 days with 500 nM palbociclib ±500 nM SNX631 or with SNX631 added later on day 7.

(F) 7-day SRB assay comparing parental and palbociclib-adapted (P-A) MCF-7 cells treated with palbociclib.

(G) 20-day SRB assay of P-A MCF-7 cells. Statistical analysis is shown in Table S1.

Data are represented as mean ± SEM.

Initial sensitivity and adaptation to palbociclib are subject to lineage plasticity, but clonal heterogeneity does not impact the effects of CDK8/19 inhibition

The known heterogeneity within the MCF-7 cell line in regard to chemotherapeutic response,[41,42] coupled with the transient nature of palbociclib adaptation seen in Figure 1G, led us to hypothesize that the adaptation to palbociclib may reflect a reversible non-genetic switch process. To study the effects of palbociclib and CDK8/19i in individual MCF-7 cells, the cells were plated at a low density (100 cells/cm^2) and analyzed by real-time video microscopy for the formation of cell clusters over 14 days. Clusters of control cells showed relatively homogeneous proliferation rates (Figure S2A, left). No clusters formed under combination treatment. The cells plated in SNX631 alone formed many clusters, similarly to control. However, the CDK8/19i eventually halted cluster proliferation and reduced cluster size, suggesting cell death (Figure S2A, middle), unlike the moderate growth inhibition seen at high density (Figure 1A). In contrast, only 10 proliferating clusters emerged under 500 nM palbociclib, a ~100-fold reduction in plating efficiency versus control. Eight of these clusters proliferated 2.75-fold slower than controls, one arrested, and one regained a control-like growth rate (Figure S2A, right). Because both compounds showed markedly greater effects at low density, we concluded

that this assay did not faithfully model palbociclib adaptation observed under high-density conditions.

To investigate the nature of palbociclib adaptation using the Luria-Delbrück fluctuation test,[7,43–45] 44 clones of the parental MCF-7 population were generated via ultra-low-density plating (Figure S2B). All clones were expanded and subjected to a 7-day concentration-response viability assay at high cell density, with dose ranges of palbociclib defined by parental IC_{50}, in the absence and in the presence of 500 nM SNX631 (Figure S3). The palbociclib IC_{50} values varied widely among the clones (Figure 2A). For some highly resistant clones, cell viability did not fall below 50% at the highest tested concentration; so, the best-fit IC_{50} values were estimated by non-linear regression. Clonal growth also varied in the absence of palbociclib but showed a little correlation with responses to palbociclib (Figure S2C), SNX631 (Figure S2D), or between the two drugs (Figure S2E). Importantly, 500 nM SNX631 potentiated the response to palbociclib in all clones, except a few that grew too slowly, for reliable analysis (Figure S3). Clone responses to 500 nM palbociclib alone and the combination were strongly correlated (r^2 = 0.82; Figure 2B), confirming that CDK8/19 inhibition augmented the antiproliferative effect of palbociclib. On the other hand, Bliss scores spanned positive, neutral, and negative values across the clones (Figure S2F), in agreement with the previous conclusion that the strong drug interaction becomes apparent only at the later time when palbociclib resistance emerges.

Variation between the clones' response to palbociclib generally increased as the concentration was increased, plateauing at approximately 60% variation for all concentrations above 63 nM (Figure S4A). Treating clonal cells with 500 nM palbociclib revealed an inter-clonal variation that was moderately higher than the noise control (variations between biological repeats of the MCF-7 parental cell line treated with 500 nM palbociclib) but statistically significant (Figure S4B). If each cell's adaptation response to CDK4/6i was purely random, we would have expected to see inter-clonal variations similar to the noise control. The non-skewed distributions of the palbociclib concentration response (Figure S4C) are consistent with the stochastic switching model of individual cells.

To confirm this conclusion, the three most palbociclib-resistant clones (18, 30, and 32) were retested twice after ~17 generations of drug-free growth between assays. All three clones showed decreased IC_{50} values in the second assay, followed by increased IC_{50} values in the third (Figure 2C). In contrast, the parental MCF-7 population showed only minor IC_{50} fluctuations, suggesting either that crosstalk between all subpopulations within MCF-7 prevents fluctuations in palbociclib sensitivity or that whatever fluctuations occur within the diverse cell lines result in regression to the mean IC_{50}.

We also performed 20-day palbociclib adaptation assays for clones 18, 30, and 32, alongside the last two IC_{50} measurements. All three clones continued proliferating in palbociclib, consistent with adaptation, but remained arrested by the addition of SNX631 (Figure 2D). Their response to the combination was even stronger than that of the parental line, indicating that Mediator kinase inhibition consistently prevents palbociclib adaptation despite variation in palbociclib sensitivity among MCF-7 subpopulations.

In vivo treatment with Mediator kinase inhibitor strongly potentiates palbociclib in palbociclib-sensitive and -insensitive ER^+ BrCa xenografts

The effects of combining palbociclib with a CDK8/19i on tumor growth *in vivo* were investigated using the MCF-7 xenograft model, which was treated for up to 80 days with vehicle control, 50 mg/kg/d palbociclib, 30 mg/kg/d SNX631-6, or a combination of the two drugs. Palbociclib significantly inhibited tumor growth, but after ~60 days of treatment, the slope of the tumor growth curve increased by up to 5-fold in the palbociclib arm (Figure 3A), indicating the development of adaptive drug resistance. SNX631-6 alone had only a moderate, albeit significant, effect on tumor growth (Figure 3A). Nevertheless, morphological analysis of the tumors collected at the endpoint indicated that 8/10 control MCF-7 tumors were highly invasive, showing tumor penetration of the muscle layer, whereas muscle invasion was detected in only 2/10 tumors treated with SNX631-6 alone (Figures 3B and S5A). These results, in concordance with similar observations on the effect of a CDK8/19i on invasive growth in a transgenic breast cancer model,[46] indicate that CDK8/19 inhibition suppresses tumor invasion, even when it has only a modest effect on the tumor growth.[47] We note that no lymph node or remote metastases were detected in our MCF7 xenograft model. Combining palbociclib with SNX631-6 drastically enhanced the effect of palbociclib, judging by the strong suppression of tumor growth (Figure 3A). In agreement with the anticipated effect of the combination, SNX631-6 potentiated palbociclib's cell-cycle arrest, as combination-treated tumors showed significantly reduced cell proliferation (shown by decreased Ki67 intensity) relative to control tumors (Figures 3C and S5B). Importantly, palbociclib, SNX631-6, and their combination neither had any effects on mouse weights (Figure 3D) nor caused other signs of toxicity in this study.

We have also tested the effects of palbociclib, SNX631-6, and their combination in another ER^+ BrCa xenograft model, T-47D. In contrast to MCF-7, T-47D cells are relatively insensitive to palbociclib and SNX631 *in vitro*, while showing a moderate response to the drug combination (Figure S5C). *In vivo*, however, both palbociclib and especially SNX631-6 had a significant effect on T-47D xenograft growth, whereas the combination of palbociclib and SNX631-6 had a much stronger effect than the individual drugs (Figure 3E), with no apparent toxicity (Figure 3F). This result indicated that the combination of CDK4/6i and CDK8/19i can produce a strong response, even in those tumors that respond weakly to either inhibitor alone.

Palbociclib adaptation is associated with phenotypic markers of senescence that are attenuated by Mediator kinase inhibition

Mechanistic analysis of the adaptation to CDK4/6 inhibition was carried out in palbociclib-treated MCF-7 cells. To elucidate the role of senescence, which is known to be induced by CDK4/6i, we carried out morphological and flow cytometric analyses during the adaptation of MCF-7 cells to 500 nM palbociclib alone or in combination with 500 nM SNX631. Palbociclib treatment induced phenotypic markers of senescence, as indicated by increased staining for senescence-associated β-galactosidase (SA-β-GAL) over the time of treatment (Figure 4A) and increases

CellPress
OPEN ACCESS

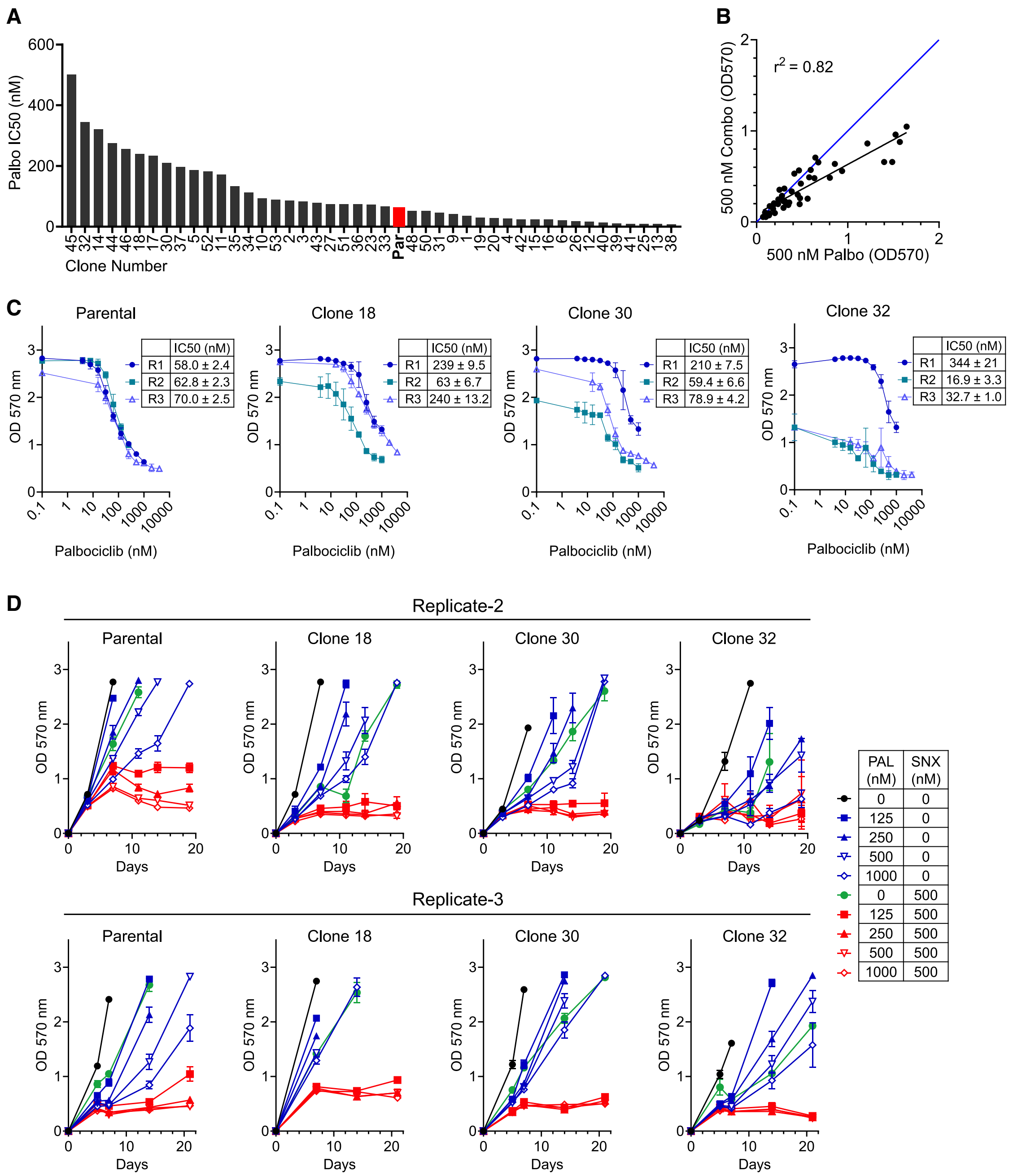

Figure 2. Effects of CDK8/19 inhibition on palbociclib adaptation in MCF-7 clones

(A) Palbociclib IC_{50} values (7-day sulforhodamine B [SRB] assays) for 44 clones (black) and the parental MCF-7 cell line (red). IC_{50} values were calculated using non-linear regression (variable slope). Best-fit IC_{50} values were estimated for clones that did not reach 50% inhibition within the dose range, but confidence intervals were undefined due to incomplete curve definition.

(B) Correlation between the effects of palbociclib and palbociclib + SNX631 across clones (7-day SRB assay). Correlation coefficients were determined by linear regression. The line of identity (x = y) is shown in blue.

(C) Dose-response curves and IC_{50} values from initial experiments (R1) and subsequent replicates (R2 and R3), for parental MCF-7 cells and three clones that were initially insensitive to palbociclib.

(D) 20-day SRB analysis of initially palbociclib-insensitive clones, from R2 (top) and R3 (bottom) (see C).

Data are represented as mean ± SEM.

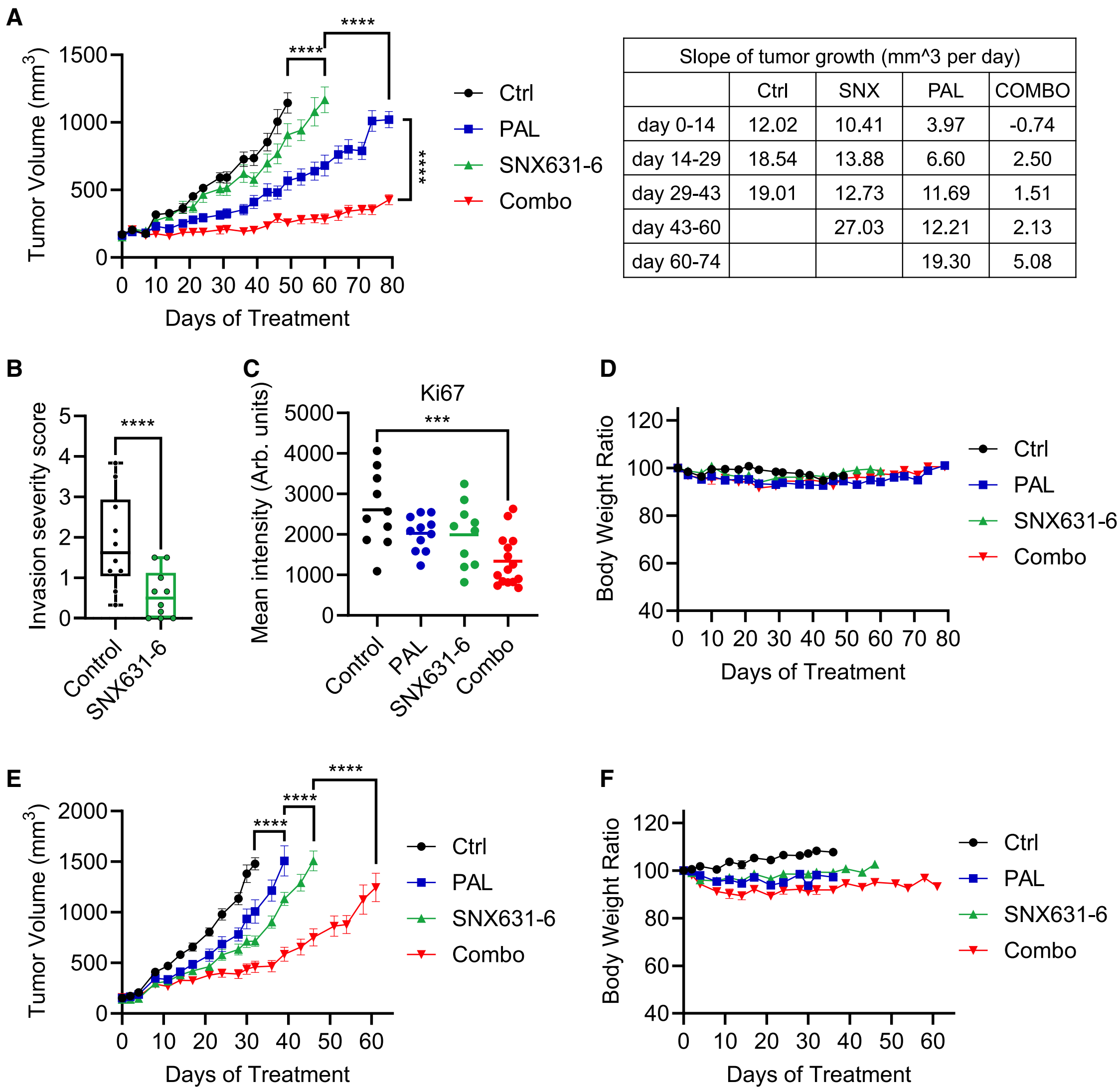

Slope of tumor growth (mm^3 per day)				
	Ctrl	SNX	PAL	COMBO
day 0-14	12.02	10.41	3.97	-0.74
day 14-29	18.54	13.88	6.60	2.50
day 29-43	19.01	12.73	11.69	1.51
day 43-60		27.03	12.21	2.13
day 60-74			19.30	5.08

Figure 3. Effects of palbociclib, SNX631-6, and their combination on tumor xenografts

(A) Tumor volume dynamics of MCF-7 xenografts (left) (n = 14 mice/group for control, palbociclib (PAL), and SNX631-6; n = 15 for the combination) and slopes of average tumor growth over indicated time intervals (right). Statistical significance was determined using mixed-effects analysis followed by Tukey's multiple-comparisons test.

(B) Histological scores for tumor invasion into muscle in vehicle- and SNX631-treated MCF-7 xenografts (n = 10 tumors per group). Tumors with multiple sections were averaged. Significance was determined using nested one-way ANOVA.

(C) Mean fluorescence intensity of Ki67 across duplicate tumor sections (n = 10 tumors per group). Significance was determined using ordinary one-way ANOVA.

(D) Body weight percentages (pretreatment weight = 100%) for the MCF-7 xenograft study.

(E) Tumor volume dynamics of T-47D xenografts (n = 10 mice/group for control, PAL, and SNX631-6; n = 9 mice for the combination).

(F) Body weight ratios for the T-47D xenograft study, normalized to mouse weight at the start of treatment.

Data are represented as mean ± SEM. ***$p < 0.001$, ****$p < 0.0001$.

in flow cytometric parameters of the forward scatter (FSC) and the side scatter (SSC), which reflect the cell size and granularity, respectively (Figure S6A). Cells that were adapted to 500 nM palbociclib over 42 days were 99% SA-β-GAL$^+$, as opposed to 10% of control cells (Figure 4B) and possessed 2- to 3-fold higher SSC and FSC values (Figure 4C). Despite these markers of the senescent phenotype, the cell number still increased in the presence of palbociclib (Figure 1G), and cell division was observed using video microscopy (Videos S1 and S2), indicating that the cells were not, in fact, senescent. This pseudo-senescent P-A cell population, while proliferating, was partially growth inhibited, as indicated by ∼10% higher fraction of cells in the G1 phase of the cell cycle (Figure S6B) and ∼60% fewer mitotic events revealed by real-time image analysis over 41.5 h (Figure S6C). The duration of mitosis itself was not significantly impacted in the P-A cells (Figure S6D).

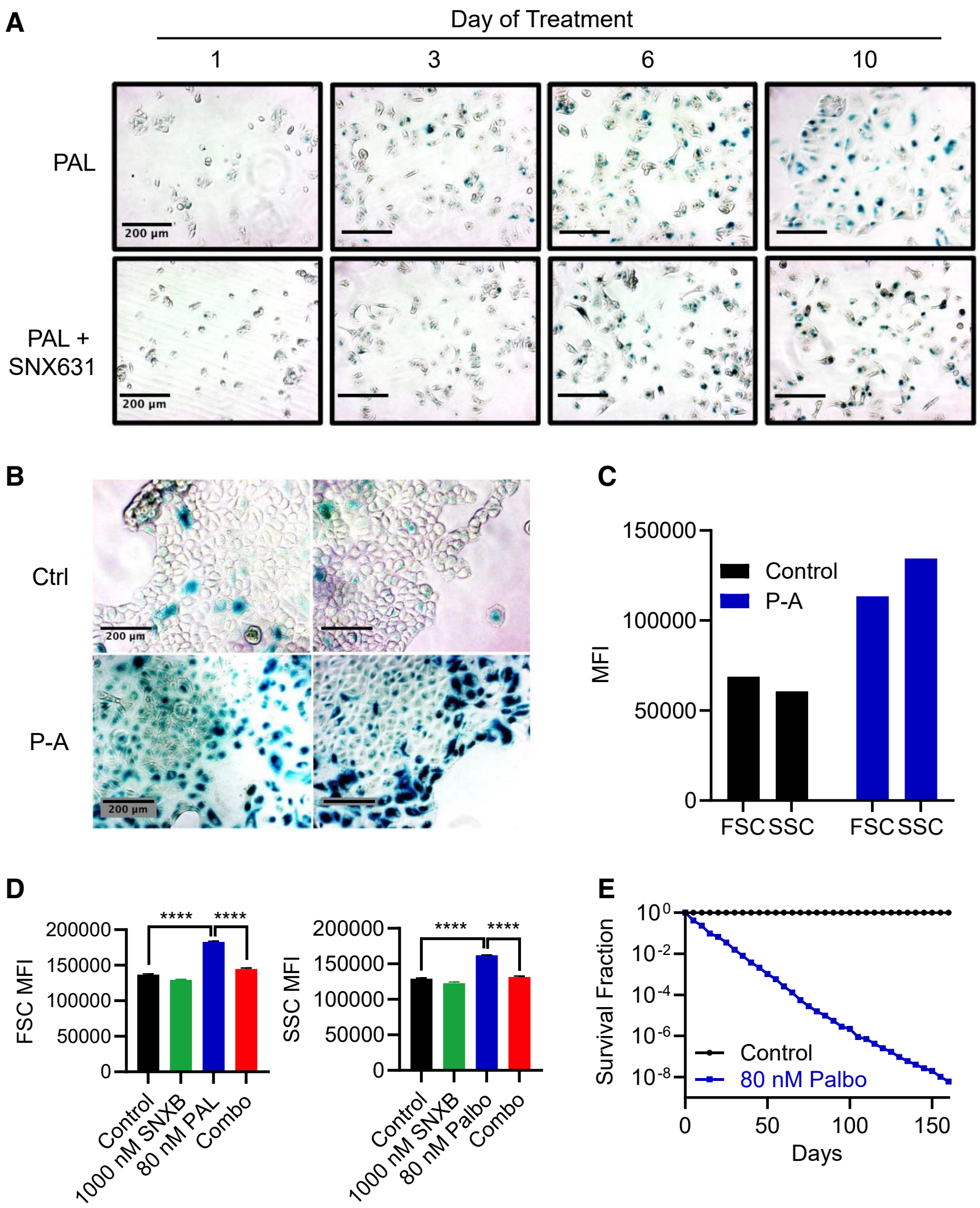

Figure 4. Effects of CDK8/19 inhibitors on phenotypic markers of senescence associated with adaptation to palbociclib

(A) Time course of MCF-7 cells treated with 500 nM palbociclib ± 500 nM SNX631 and stained for SA-β-GAL (green) (scale bars, 200 μm).

(B) SA-β-GAL-stained MCF-7 cells treated with 0.1% DMSO vehicle control or 500 nM palbociclib for 42 days (2 representative images each) (scale bars, 200 μm).

(C) Forward scatter (FSC) and side scatter (SSC) of control (black) and 42-day palbociclib-adapted (blue) cells, measured as mean fluorescence intensity (MFI).

(D) FSC and SSC of MCF-7 cells following 5 days of treatment with palbociclib ± senexin B (SNXB). Statistical significance was determined by ordinary one-way ANOVA with Sidak's multiple-comparisons test (assuming a single pooled variance). $^{****}p < 0.0001$.

(E) MCF-7 cells were treated with 80 nM palbociclib or DMSO vehicle control for 160 days. Cells were trypsinized, counted, and replated every 5 days. Data are represented as mean ± SEM.

The addition of SNX631 to palbociclib did not affect the percentage of SA-β-GAL$^+$ cells (Figure 4A), but CDK8/19i senexin B fully suppressed the increase in FSC (a measure of the cell size) and SSC (a measure of granularity) in the palbociclib-treated cells (Figure 4D), indicating a role for Mediator kinases in some but not all phenotypic features of the pseudo-senescent phenotype.

A positive role of the pseudo-senescent phenotype in palbociclib adaptation was indirectly suggested by the results of an experiment where MCF-7 cells were placed under a relatively low (80 nM) concentration of palbociclib, and their growth was assessed every 5 days following trypsinization and replating (rather than in constant presence of the drug). The 80 nM palbociclib-treated population showed a consistently reduced growth rate throughout the 160 days of the experiment, without developing resistance (Figure 4E). This result suggests that maintaining cells under the drug without frequent replating was critical for their adaptation to palbociclib, as can be seen in Figure 1. Notably, cells displaying a pseudo-senescent phenotype are not easily dissociated by trypsinization and are preferentially lost during replating, suggesting that the loss of such cells could be responsible for the failure of palbociclib adaptation in this experimental setup.[48]

The effects of the combination of CDK4/6 and Mediator kinase inhibitors remain dependent on Rb

Because CDK4/6 acts through Rb phosphorylation and CDK4/6i require Rb for their antiproliferative activity, we investigated the effects of palbociclib/SNX631 combination on Rb and its interactive proteins (cyclin E1 and E2F1) through western blotting analysis in MCF-7, ZR-75-1, and SW-620 cells (Figure 5A). Approximately 1 week of treatment with palbociclib led to a decrease in total Rb levels in all three cell lines and decreased Rb phosphorylation, especially at S780. The decrease in Rb levels was even more pronounced in the cells treated with the palbociclib and SNX631 combination. Consistent with previous studies on palbociclib adaptation/resistance,[15] 7 days of treatment with palbociclib increased the expression of cyclin E1 in all three cell lines; the addition of SNX631 did not notably alter this effect of palbociclib. SNX631 and palbociclib moderately decreased E2F1 levels individually, especially in MCF-7 and SW-620 cells, and the combination exacerbated this decrease (Figure 5A).

The enhancement of the decrease in total Rb levels was the strongest effect of the addition of the Mediator kinase inhibitor to palbociclib in the above analysis. To determine if this effect reflected a direct impact of the drug combination on Rb or reflected a more robust selection for cells with lower Rb expression (a common mechanism of resistance to CDK4/6i), we generated

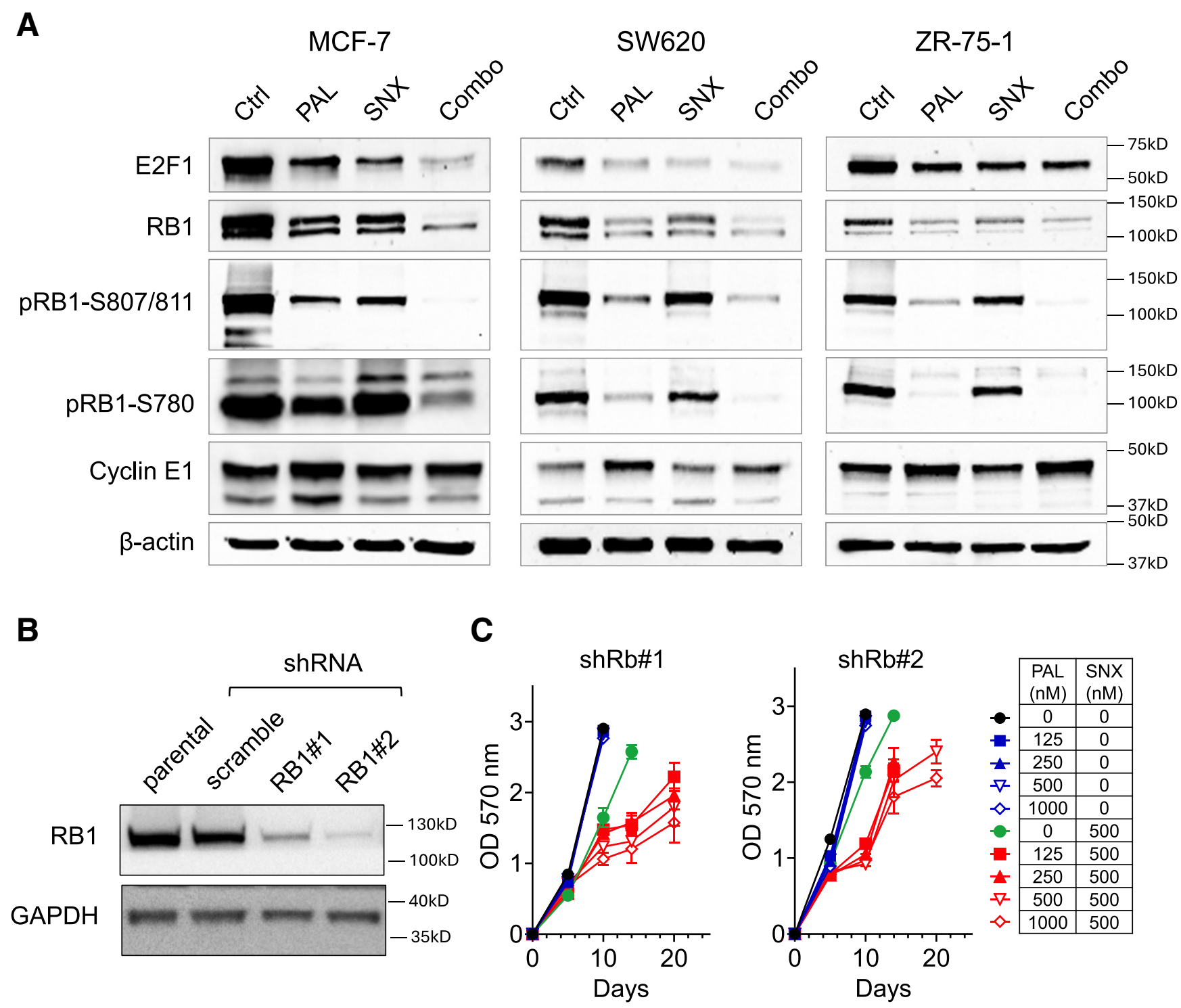

Figure 5. Effects of palbociclib and CDK8/19 inhibitors on Rb and related proteins

(A) Western blot analysis of the indicated proteins in MCF-7, ZR-75-1, and SW-620 cell lines treated with 500 nM palbociclib (PAL) ± 500 nM SNX631 (SNX) for 6–7 days.

(B) Western blot of two Rb-knockdown derivatives (shRNA-RB#1 and #2) of the MCF-7 cell line.

(C) 20-day SRB assays of shRNA-RB#1 and #2 treated with palbociclib with (red) or without (blue) 500 nM SNX631. 500 nM SNX631 monotherapy is in green. Data are represented as mean ± SEM.

two MCF-7 derivatives with short hairpin RNA (shRNA)-mediated RB1 knockdown (Figure 5B). These derivatives were then tested for their response to different concentrations of palbociclib alone or in combination with SNX631 over 20 days (Figure 5C). In contrast to the parental MCF-7 cells (Figure 1A), the Rb-knockdown derivatives were no longer inhibited by palbociclib alone (Figure 5C). The moderate antiproliferative effect of SNX631 alone was undiminished by RB1 knockdown, but the combination of palbociclib and SNX631, while more potent than palbociclib alone, no longer produced complete suppression of cell proliferation that was seen in the parental cells. Hence, the antiproliferative effect of the combination of CDK4/6 and Mediator kinase inhibitors remains partially reliant on Rb, resulting in enhanced selection of cells with Rb downregulation upon treatment with the drug combination.

RNA sequencing analysis identifies transcriptomic changes associated with palbociclib adaptation that are prevented by CDK8/19 inhibition

To investigate transcriptomic changes underlying palbociclib adaptation and its prevention by CDK8/19 inhibition, we conducted short-term (ST) and long-term (LT) *in vitro* RNA sequencing (RNA-seq) studies in MCF-7 cells and an *in vivo* (IV) study of the xenografts shown in Figure 3A (Figure 6A). The ST study analyzed cells after 72 h of treatment with vehicle, 500 nM SNX631, 500 nM palbociclib, or their combination. The LT study compared ten independently adapted populations treated with 500 nM palbociclib for 28 days with five vehicle-treated controls; this study did not include the addition of SNX631 because no cells remained after 28 days of combination treatment. The IV study analyzed tumors from 4–5 mice per group after nearly 80 days of treatment with each drug or drug combination to assess palbociclib resistance and its prevention by SNX631-6.

Gene set enrichment analysis (GSEA) identified enriched hallmark gene sets in the ST and LT studies (Figure 6B) and separately analyzed human (tumor) and mouse (stromal) genes in the IV study (Figures 6C and 6D). The most common response to palbociclib upon ST treatment or LT adaptation *in vitro* and in *in-vivo*-adapted tumor or stromal cells was the downregulation of the MYC targets (V1), and the downregulation of this pathway was reduced by the addition of CDK8/19i (Figures 6B and 6C; Figure S7A). Overall, however, most of the pathways affected by palbociclib treatment in tumor cells during the adaptation *in vitro* (LT) or IV were upregulated. Remarkably, although IV treatment with SNX631-6 alone had a very similar effect to palbociclib on most pathways in tumor (but not in stromal) cells, the addition of CDK8/19i to palbociclib decreased the upregulation of almost all pathways (Figures 6C and 6D), in agreement with the principal role of Mediator kinases as regulators of transcriptional reprogramming induced by other agents.[49] IFN-γ and IFN-α pathways were the most strongly upregulated in P-A tumor cells *in vivo*, followed by TNF-α-NF-κB signaling, and were also strongly induced during LT adaptation *in vitro* but not ST treatment or in stromal cells (Figures 6B–6D; Figures S7B–S7D). These results are concordant with the study[50] where upregulation of the IFN-γ, IFN-α, and TNF-α signaling pathways, resulting from the activation of retrotransposons carrying short, interspersed repeat sequences that activate cGAS-STING pathway, was implicated in the acquisition of drug resistance in mammary carcinoma. Our RNA-seq analysis was limited to poly(A)$^+$ RNA and, therefore, did not provide information on the repeated sequences, but we analyzed the effects of *in vivo* treatments on the cGAS-STING pathway and found that it was upregulated in P-A tumor cells (Figure S7E), in agreement with the possible retrotransposon activation.[50] The addition of CDK8/19i decreased the induction of these pathways during palbociclib adaptation (Figures 6B and 6C; Figure S7). Other prominent effects of the drug combination relative to palbociclib alone were associated

CellPress
OPEN ACCESS

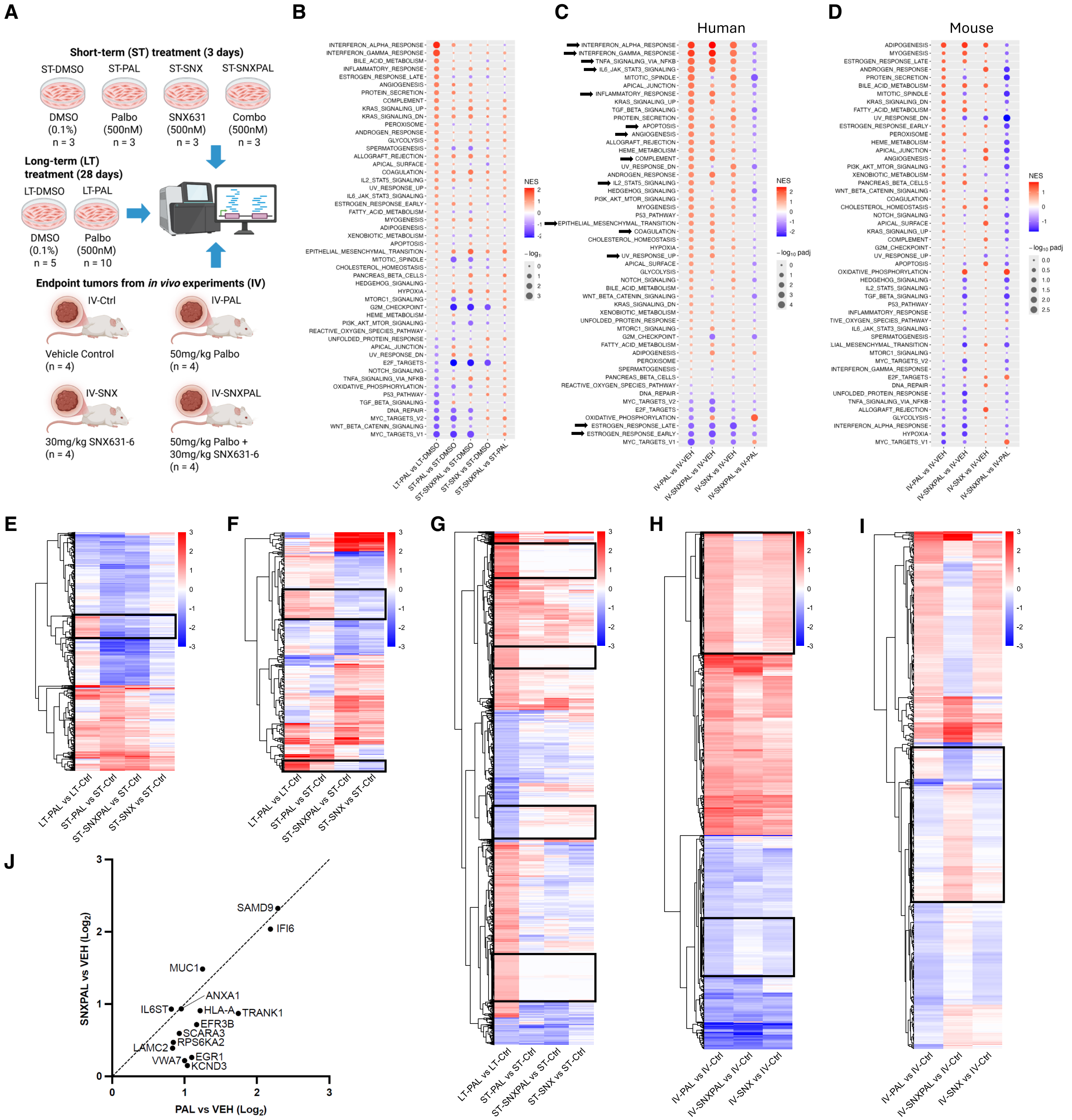

Figure 6. RNA-seq analysis of palbociclib resistance and its prevention by CDK8/19 inhibition

(A) Schematic of three RNA-seq experiments in MCF-7 cells: short-term (ST; 3-day) and long-term (LT; 28-day) *in vitro* treatments, and an *in vivo* (IV) analysis of excised MCF-7 tumors. For LT-treated cells, drug-containing medium was replaced every 5 days. Vehicle control cells were passaged every 7 days, and palbociclib-treated cells were passaged once on day 10 to prevent overgrowth.

(B–D) Normalized enrichment scores (NESs) from GSEA hallmark pathways significantly affected in any treatment comparison *in vitro* (B) or *in vivo* human tumor (C) and stromal mouse (D) compartments. Black arrows indicate pathways implicated in CDK4 inhibitor resistance in a study by Palmer et al.[5]

(E–I) Heatmaps showing average $\log_2$ fold change relative to corresponding control samples with unsupervised hierarchical clustering. Differentially expressed genes (DEGs) were identified for the following comparisons: ST-PAL vs. ST-DMSO (711 DEGs) (E), ST-SNXPAL vs. ST-PAL (273 DEGs) (F), LT-PAL vs. LT-DMSO (3,599 DEGs) (G), IV-PAL vs. IV-VEH (1,023 DEGs) (H), and IV-SNXPAL vs. IV-PAL (515 DEGs) (I). (The designations of the experimental groups are shown in A). Black boxes highlight gene expression patterns of interest. DEGs were selected using a fold change >1.5 and FDR < 0.05. Heatmaps were generated using the Morpheus platform.

(J) Plot of $\log_2$ fold change for IV-SNXPAL and IV-PAL relative to IV-VEH for the 14 DEGs that showed >2-fold induction in atirmociclib-resistant versus atirmociclib-sensitive tumors and were also induced by IV-PAL.

with the oxidative phosphorylation pathway, which was upregulated by the combination treatment both *in vitro* (ST) and especially *in vivo* (in both tumor and stromal cells), and the mitotic spindle pathway, which was downregulated by the combination, in agreement with its antiproliferative effect (Figures 6B and 6C).

At the level of individual genes, differentially expressed genes (DEGs) between any treatment conditions were defined as genes with >1.5-fold change and a false discovery rate (FDR) of < .05. Heatmap visualization of the 711 DEGs affected by palbociclib in the ST *in vitro* study (ST-PAL) (see Data S1) showed that some were similarly affected by the ST and the LT palbociclib treatment, but many others were no longer affected in the LT (P-A) cells or even affected in the opposite direction (bracketed in Figure 6E; heatmaps for individual samples are shown in Figure S8A). Observation of the 273 DEGs identified by ST-SNXPAL versus ST-PAL comparison revealed clusters of genes where SNX631 prevented the same ST palbociclib-induced expression patterns that were exacerbated by LT palbociclib treatment (bracketed in Figure 6F). Among 3,599 DEGs affected in P-A cells (LT-PAL), only a minority were already affected after ST-PAL treatment, indicating that most of the transcriptional changes in LT-adapted cells developed late in the course of the adaptation (bracketed in Figures 6G and S8B).

Of note, all 10 independently adapted LT populations showed a very similar pattern of gene expression for both the ST and LT DEGs (Figure S8A and S8B), indicating that either all 10 populations underwent the same transcriptomic changes during their adaptation or the adaptation represented the selection of the same intrinsically resistant subpopulation of MCF-7 cells.[44] The latter possibility would be in contrast to the conclusions of the fluctuation analysis (Figure S4). We used the published data from single-cell RNA sequencing (scRNA-seq) analysis of 977 MCF-7 cells[51] to determine if the changes in gene expression associated with LT adaptation could be found among the naive cells. We compared 42 significantly expressed LT DEGs across 977 single cells with the LT-PAL and LT-Ctrl samples (Figure S9). For nearly all 42 genes, single cells' expression values clustered close to each other, regardless of the similarity of expression to the LT-PAL samples. The absence of individual outlier cells that resembled LT-PAL gene expression (with the possible exception of two genes, *HMGN5* and *IFIT1*) suggests independent, similar adaptation mechanisms of different populations, rather than pre-selection of inherently resistant cells.

In the IV study, analysis of the 1,023 tumor DEGs identified in palbociclib-resistant tumors (IV-palbo versus IV-vehicle) revealed clusters of gene changes, which were counteracted by the addition of SNX631-6 (bracketed in Figures 6H and S8C) and may include gene candidates for the prevention of palbociclib resistance by CDK8/19i treatment. Interestingly, a subset of 515 DEGs identified by the comparison of IV-combo versus IV-palbo also showed combination-specific expression changes (bracketed in Figures 6I and S8D). The effects of different treatments on gene expression were largely unaffected by the intra-treatment group size of tumors upon collection (Figures S8C and S8D), suggesting similar mechanisms of IV palbociclib adaptation and its reversal by CDK8/19 inhibition in different tumors.

A recent study[5] identified the pathways and genes affected by the development of *in vivo* resistance to a CDK4-selective inhibitor, atirmociclib, in another ER^+ BrCa xenograft model, ZR75-1. Remarkably, 12 of 14 pathways identified as being upregulated in atirmociclib-resistant tumors in that study were also upregulated in palbociclib-resistant MCF-7 xenografts (arrows in Figure 6C), and the upregulation of all 12 pathways was decreased by the combination of atirmociclib with the CDK8/19i (Figure 6C). In the same study,[5] 129 genes with >2-fold induction in their CDK4 inhibitor-resistant versus CDK4 inhibitor-sensitive ZR75-1 xenografts were identified. Among these, 14 were also found to be induced in palbociclib-treated MCF-7 tumors (Figure 6J). Of those 14 genes implicated in *in vivo* resistance to different CDK4 inhibitors in different tumor models, 11 exhibited suppression of their induction by combination treatment (Figure 6J).

At the level of individual tumor genes, *CDK8* expression remained largely unchanged upon palbociclib adaptation both *in vitro* and *in vivo*, whereas *CDK19* expression increased following palbociclib adaptation *in vivo* (but not *in vitro)*, and this increase was diminished by the addition of the CDK8/19i (Figure 7A). In contrast, *CDK4* expression was consistently reduced by palbociclib, and this downregulation was unaffected by the addition of CDK8/19i (Figure 7A). Figure 7B shows genes that were consistently upregulated in P-A cells both *in vivo* and *in vitro* LT, and where this *in vivo* induction was decreased by the addition of the CDK8/19i (Figure 7B). The first gene, *CDK6*, is frequently amplified or upregulated in cells resistant to CDK4/6i. *CDK6* was upregulated by palbociclib treatment both in ST and LT *in vitro* studies; the addition of CDK8/19i suppressed *CDK6* induction both *in vitro* and *in vivo*. The same *in vitro* and *in vivo* expression pattern was observed for *NBEA*. The other genes in this group, *SLC12A7* (reported to promote tumor invasion)[52] and *VWA7* (a marker of *in vivo* resistance to atirmociclib),[5] were significantly upregulated *in vitro* only in LT but not in ST. Furthermore, their induction *in vivo* was suppressed by SNX631-6.

We also observed a select group of genes that were downregulated during *in vitro* LT adaptation, and these genes also possessed the pattern of *in vivo* palbociclib upregulation mediated by the palbociclib + SNX631-6 combination (Figure 7C). Three of these genes were upregulated by ST palbociclib treatment, including *RAB11FIP4*, reported to promote the cancer stem cell phenotype,[49] and *OXTR*, which induces mammary carcinogenesis[53]; the ST induction of these genes was decreased by the addition of SNX631. The third gene, *TGFB2*, implicated in breast cancer progression,[54] was also induced by palbociclib in the ST study, but the addition of SNX631 increased its induction even further. The fourth gene, *EGR1*, a transcription factor associated with atirmociclib resistance,[5] was downregulated by palbociclib both in the ST and the LT study (and further downregulated by the CDK8/19i combination in ST).

The expression of several genes was affected in tumors treated by the drug combination but relatively unchanged in tumors treated with the individual drugs (Figure 7D). Interestingly, while these genes were unaffected in palbociclib-resistant tumors, they were all affected (downregulated or upregulated) by

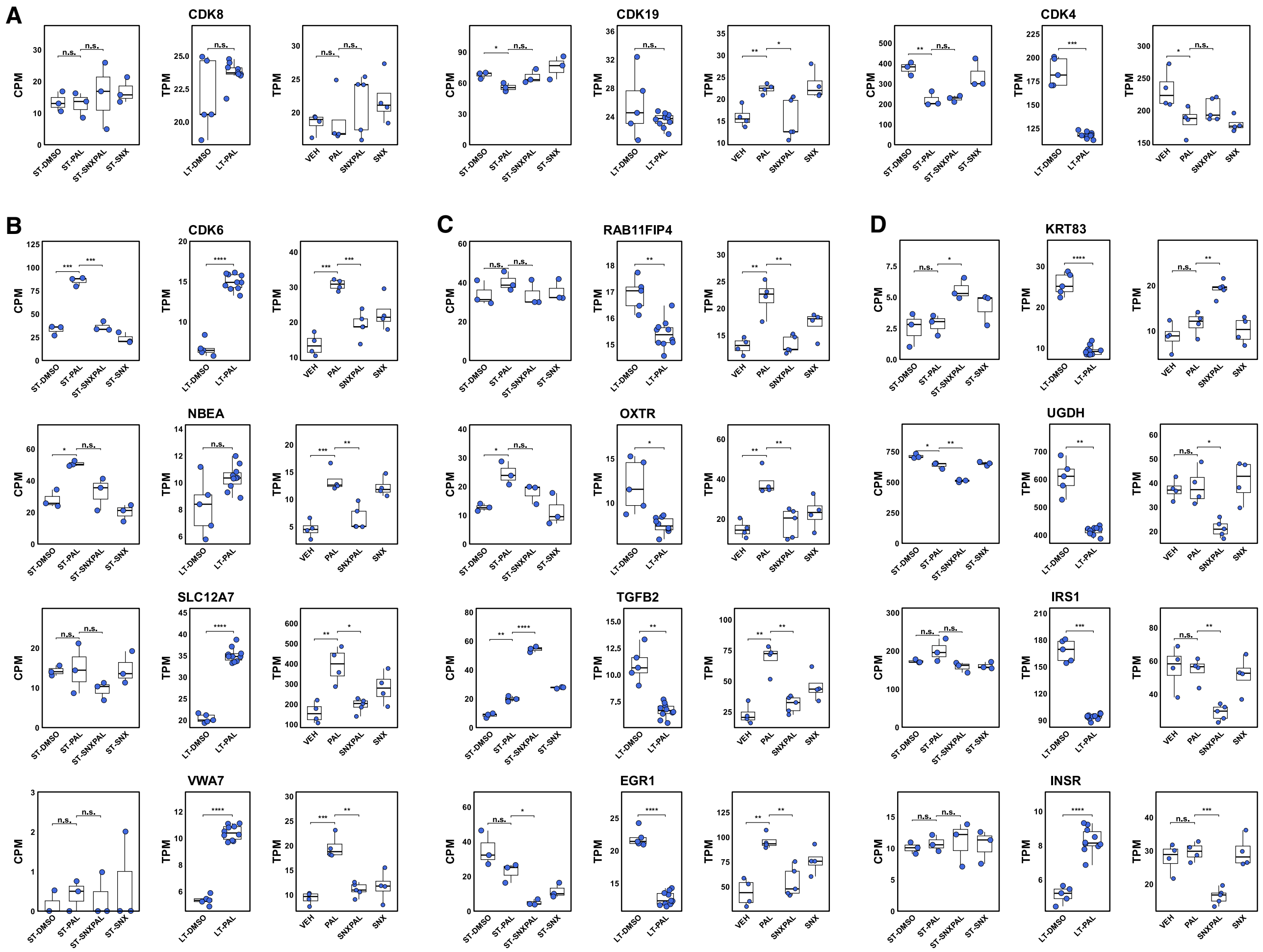

Figure 7. RNA-seq analysis of palbociclib resistance and its prevention by CDK8/19 inhibition

(A–D) Box-and-whisker plots of selected genes of interest. (A) Expression of *CDK8*, *CDK19*, and *CDK4* in RNA-seq samples from the *in vitro* and *in vivo* datasets. (B and C) Genes overexpressed *in vivo* in IV-palbo and suppressed in IV-combo, where this overexpression was either concordant (B) or non-concordant (C) with *in vitro* adaptation (LT-palbo).

(D) Genes showing strong upregulation or downregulation in LT-PAL, with combination-specific effects observed *in vivo*. Boxes represent the interquartile range (IQR), the horizontal line indicates the median, whiskers extend to the most extreme observations within 1.5× IQR, and overlaid points represent individual biological replicates.

Gene expression values (CPM [counts per million] for *in vitro* and TPM [transcripts per million] for *in vivo* datasets) were compared between selected treatment groups, using two-tailed Welch's t tests (unpaired, unequal variances assumed) performed separately for each gene and comparison. *$p < 0.05$, **$p < 0.01$, ***$p < 0.001$, and ****$p < 0.0001$.

LT palbociclib adaptation *in vitro*. The first gene, *KRT83*, is one of several keratin genes that were upregulated by the drug combination, similarly to multiple keratin family members that were upregulated by CDK8/19 inhibition in combination with castration in castration-resistant prostate cancers.[36] The other three genes were downregulated by the drug combination and, remarkably, all three have been reported to have broad tumor-promoting activities in breast and other cancers. These include *UGDH*,[55] *IRS1*,[56] and *INSR*.[57]

Hence, prevention of adaptive palbociclib resistance by CDK8/19 inhibition appears to involve both the suppression of transcriptional reprogramming associated with the development of resistance and downregulation of several tumor-supporting genes by the combination of CDK4/6i and CDK8/19i.

DISCUSSION

In the present study, we found that the adaptive resistance to different CDK4/6i rapidly develops *in vitro* and *in vivo*, with similar rapid kinetics in different ER^+ BrCa and other tumor cell lines. Luria-Delbrück fluctuation analysis showed fluctuating short-term palbociclib sensitivity among 44 MCF-7 clones, with no resistant outliers indicative of a heritable pre-resistant phenotype. Furthermore, both mass populations and individual clones lost resistance after drug withdrawal, supporting a transient, non-genetic mechanism. Ten independently adapted MCF-7 populations exhibited remarkably similar transcriptomic changes. This similarity most likely reflects concordant transcriptional reprogramming, rather than a selection of stable

pre-existing variants, because scRNA-seq data from 977 untreated MCF-7 cells revealed no drug-naive cells with transcriptomic markers of palbociclib adaptation.

Adaptive palbociclib resistance *in vitro* was associated with a pseudo-senescent phenotype. Despite displaying senescence markers, adapted cells continued to divide, although with delayed G1 progression and fewer mitoses, potentially reflecting downregulation of the MYC pathway. Frequent replating, which preferentially removes highly adherent cells with senescent morphology, prevented adaptation during prolonged palbociclib treatment. This finding suggests that senescent-like cells contribute to adaptation, possibly through paracrine SASP effects.[22] Remarkably, interferon signaling is known to be a major trigger of SASP,[58] and interferon pathways were drastically upregulated in cells that were adapted to palbociclib *in vitro* and *in vivo*. Specifically, IFN-γ, IFN-α, and TNF-α signaling via NF-κB were the top three most upregulated pathways by palbociclib *in vivo*. A possible cause of the upregulation of these pathways could be the activation of retrotransposons carrying repeat sequences that activate cGAS-STING, leading to interferon pathway activation and the acquisition of drug resistance.[50] Our RNA-seq analysis was carried out on poly(A)$^+$ RNA and, therefore, could not be used to analyze the repeatome components, but we found that the cGAS-STING pathway was activated in P-A xenografts, in accordance with the retrotransposon activation hypothesis.

Given the known role of transcriptional changes in the resistance to CDK4/6i, we tested if such resistance would be prevented by the inhibition of CDK8/19 Mediator kinases, pleiotropic regulators of transcriptional reprogramming. In contrast to pleiotropic transcriptional inhibitors, such as inhibitors of CDK7 and CDK9, CDK8/19i alone had only limited and variable antiproliferative effects, and their combination with CDK4/6i showed no reproducible evidence of synergistic or even additive effects in shorter-term (7-day) studies. However, CDK8/19i prevented the development of CDK4/6i resistance in prolonged assays, a highly specific effect of CDK8/19 inhibition. The prevention of resistance was observed in different cell lines, both in drug-naive and in P-A cells, and in all MCF-7 clones with different levels of palbociclib sensitivity. The antiproliferative effects of CDK4/6i and CDK8/19i combination, like the effects of CDK4/6i alone, remained dependent on Rb, as demonstrated by the more rapid loss of Rb in cells treated with the drug combination and by reduced sensitivity to this combination in cells with Rb knockdown.

Treatment with the CDK8/19i also potentiated the effects of CDK4/6i *in vivo*, with the effects of the addition of CDK8/19i SNX631-6 consistent with the prevention of palbociclib resistance in MCF-7 xenografts, where the effect of CDK4/6i alone was initially strong, whereas the CDK8/19i alone had only a modest effect. Remarkably, long-term synergy between palbociclib and SNX631-6 was observed even in xenografts formed by T-47D cells, which were insensitive to either drug *in vitro*, although both of the individual drugs, especially SNX631-6, had a significant effect on these cells *in vivo*, in agreement with the preferential *in vivo* sensitivity to CDK8/19i observed in triple-negative breast cancer.[34]

Most of the transcriptomic changes in cells adapted to palbociclib *in vitro* arose late, limiting the analysis of the effects of the drug's combination with CDK8/19i, as the combination-treated cells did not survive long enough for analysis. However, we were able to analyze the transcriptomic effects of the drug combination on the development of long-term palbociclib resistance *in vivo*. The addition of the CDK8/19i to palbociclib had two major effects at the level of pathways and individual genes. The first effect was reduced upregulation of the genes and pathways associated with tumor growth in the presence of palbociclib. This was observed for several pathways that regulate gene expression, such as TGF-β signaling, hedgehog signaling, Notch signaling, and Wnt/β-catenin signaling, as well as the cGAS-STING pathway, a probable upstream inducer of the interferon pathways. Remarkably, the addition of a CDK8/19i decreased the upregulation of all 12 pathways that were upregulated both in palbociclib-treated MCF-7 xenografts and the ZR75-1 xenografts treated with a selective CDK4 inhibitor, atirmociclib.[5] Several of these pathways, including TNF-α-NF-κB signaling, IL-6-JAK-STAT3 signaling, inflammatory response, angiogenesis, epithelial-mesenchymal transition, and estrogen response, have been previously reported to be positively regulated by CDK8/19.[25,26,46,59–61] At the individual-gene level, CDK8/19i suppressed palbociclib-induced CDK6 expression both *in vitro* and *in vivo*, as well as the induction of several other tumor-promoting or CDK4/6i resistance-associated genes, including *SLC12A7*, *RAB11FIP4*, *OXTR*, *EGR1*, and *VWA7*, indicating that the inhibition of CDK8/19-mediated transcriptional reprogramming affects multiple markers and mechanisms of resistance to CDK4/6-targeting drugs. The second major effect was specific to the drug combination and included induction of keratin genes and the oxidative phosphorylation pathway, together with suppression of the mitotic-spindle and G2/M-checkpoint pathways associated with cell proliferation. Remarkably, individual genes that were most strongly and selectively downregulated by the drug combination, such as *UGDH*, *IRS1*, and *INSR*, have known tumor-promoting activities. Hence, CDK8/19i offer long-term potentiation of CDK4/6-targeting drugs both by interfering with transcriptional reprogramming associated with tumor cell adaptation to the latter drugs and by the specific effects of the CDK4/6 and CDK8/19i combination on genes that regulate tumor growth.

These findings support the therapeutic potential of combining inhibitors of CDK4/6 and CDK8/19. CDK4/6i are used for the treatment of ER$^+$ BrCa in combination with antiestrogens, and the addition of CDK8/19i to this combination is likely to be especially beneficial, given the known co-operative effect of CDK8/19i and antiestrogens.[26] CDK4/6i are also being actively investigated for the treatment of other malignancies, where their combination with CDK8/19i is also likely to be beneficial.

Limitations of the study

Our study has several limitations. First, although we observed consistent effects across multiple established breast cancer cell line models, these systems do not fully recapitulate the heterogeneity of human tumors. Future studies using patient-derived xenografts and organoid models are important to further evaluate the translational potential of CDK8/19 inhibitors in combination with CDK4/6 inhibitors. Second, while our studies revealed the ability of CDK8/19 inhibitor to suppress

transcriptional reprogramming associated with the adaptation to palbociclib *in vivo*, we were unable to perform long-term transcriptomic analyses of the CDK4/6-CDK8/19 inhibitor combination *in vitro* because the strong efficacy of the combination largely prevented the emergence of adapted cell populations. In addition, our *in vivo* efficacy studies were performed in NSG mice and, therefore, did not permit assessment of tumor-immune interactions. Future studies in immunocompetent models are needed to determine how CDK8/19 inhibition affects CDK4/6 inhibitor-induced remodeling of the breast tumor microenvironment, antitumor immunity, and therapeutic response. Finally, because our RNA-seq analysis was restricted to poly(A)$^+$ transcripts, we were unable to comprehensively analyze the repeatome or directly assess retrotransposon activation. Future studies employing total RNA-seq, long-read sequencing, or dedicated repeatome analyses are necessary to determine whether activation of transposable elements contributes to the interferon and cGAS-STING signatures observed during adaptation.

RESOURCE AVAILABILITY

Lead contact

Requests for further information and resources should be directed to and will be fulfilled by the lead contact, Mengqian Chen (chenm@cop.sc.edu).

Materials availability

This study did not generate new unique reagents.

Data and code availability

- RNA-seq data have been deposited in Gene Expression Omnibus as GEO: GSE303026 and are publicly available as of the date of publication.
- This paper analyzes existing, publicly available data, accessible on the NCBI Gene Expression Omnibus at GEO: GSE144320 (MCF-7 scRNA-seq).
- All data reported in this paper will be shared by the lead contact upon request.
- This paper does not report original code.
- Any additional information required to reanalyze the data reported in this paper is available from the lead contact upon request.

ACKNOWLEDGMENTS

We thank Kayla Poe, Stephan Bowe, Bailey Kane, Emma Markwell, Ho Yen, Sanjana Tripuraneni, Nikitha Sashi, Samantha Safa, Rebekah Martin, Marina Ostanin, Linda Cui, and William Michel for their assistance with experimentation. We thank Dr. Michael Shtutman and the Functional Genomics Core of the COBRE Center for Targeted Therapeutics at the University of South Carolina (USC) supported by NIH grant P20GM109091. This work was supported by NIH grants R01CA266027 (I.B.R., M.C, and E.V.B.), R43CA271996 (M.C. and E.V.B.), R44CA203184 (M.C. and I.B.R.), P20GM109091 (E.V.B. and I.B.R.), and SPARC Graduate Research Grants, USC (X.D., J.L., and Z.T.M.). A.S. acknowledges the support of NIH-NIGMS via grant R35GM148351. M.S.A., A.K., and V.T. were supported by the Ministry of Science and Education of the Russian Federation, project no. 075-15-2025-475, and they acknowledge IGB RAS facilities supported by the Ministry of Science and Higher Education of the Russian Federation for providing equipment of carry out the experiments.

AUTHOR CONTRIBUTIONS

Conceptualization, E.V.B., I.B.R., M.C., V.T., and A.S.; methodology, Z.T.M., M.Y., A.K., A.C.S., X.D., J.L., K.D., A.E., E.B., G.M., M.S.-A., G.C., T.A.H., C.C., G.P.S., V.S., C.-u.L., A.S., V.T., and M.C.; investigation, Z.T.M., A.S., M.C., E.V.B., and I.B.R.; writing – original draft, Z.T.M., E.V.B., and I.B.R.; writing – review & editing, Z.T.M., E.V.B., M.C., and I.B.R.; funding acquisition, E.V.B., M.C., and I.B.R.; resources, E.V.B. and I.B.R.; supervision, E.V.B., M.C., and I.B.R.

DECLARATION OF INTERESTS

M.C. is director of research; I.B.R. is president and chief scientific officer; T.A.H. is a former employee; and E.V.B., J. L., and Z.T.M. are consultants of Senex Biotechnology, Inc.; I.B.R., E.V.B., M.C., and T.A.H. are stockholders of Senex Biotechnology, Inc.; I.B.R., M.C., and J.L. are inventors of US patent 11,572,369; E.V.B., I.B.R., X.D., T.A.H., and Z.T.M. are inventors of patent application PCT/US2022/043852.

STAR★METHODS

Detailed methods are provided in the online version of this paper and include the following:

- KEY RESOURCES TABLE
- EXPERIMENTAL MODEL AND STUDY PARTICIPANT DETAILS
  - Cell lines
  - Mice
- METHOD DETAILS
  - Cell proliferation assays
  - Bliss synergy analysis
  - Senescence-associated-B-galactosidase staining
  - Cell cloning
  - Time-lapse video microscopy
  - RNA-SEQ
  - Lentiviral knockdown of RB
  - Western blot
  - Analysis of publicly available RNA-seq data
  - Immunofluorescence (IF) labeling, H&E staining, and analysis
- QUANTIFICATION AND STATISTICAL ANALYSIS

SUPPLEMENTAL INFORMATION

Supplemental information can be found online at https://doi.org/10.1016/j.xcrm.2026.103004.

Received: August 22, 2025
Revised: March 22, 2026
Accepted: July 28, 2026
Published: August 19, 2026

REFERENCES

1. Rubin, S.M., Sage, J., and Skotheim, J.M. (2020). Integrating old and new paradigms for G1/S control. Mol. Cell *80*, 183–192. https://doi.org/10.1016/j.molcel.2020.08.020.

2. Gao, T., Sun, Y., Leng, P., Liu, D., Guo, Q., and Li, J. (2025). CDK4/6 inhibitors in breast cancer therapy: mechanisms of drug resistance and strategies for treatment. Front. Pharmacol. *16*, 1549520. https://doi.org/10.3389/fphar.2025.1549520.

3. Guo, Z., Dong, R.W., Wu, Y., Dong, S., and Alahari, S.K. (2025). Cyclin-dependent kinase 4 and 6 inhibitors in breast cancer treatment. Oncogene *44*, 1135–1152. https://doi.org/10.1038/s41388-025-03378-0.

4. Luo, L., Yang, P., Mastoraki, S., Rao, X., Wang, Y., Kettner, N.M., Raghavendra, A.S., Tripathy, D., Damodaran, S., Hunt, K.K., et al. (2025). Single-cell RNA sequencing identifies molecular biomarkers predicting late progression to CDK4/6 inhibition in patients with HR+/HER2- metastatic breast cancer. Mol. Cancer *24*, 48. https://doi.org/10.1186/s12943-025-02226-9.

5. Palmer, C.L., Boras, B., Pascual, B., Li, N., Li, D., Garza, S., Huser, N., Yuan, J.T., Cianfrogna, J.A., Sung, T., et al. (2025). CDK4 selective inhibition improves preclinical anti-tumor efficacy and safety. Cancer Cell *43*, 464–481.e14. https://doi.org/10.1016/j.ccell.2025.02.006.

6. Marine, J.-C., Dawson, S.-J., and Dawson, M.A. (2020). Non-genetic mechanisms of therapeutic resistance in cancer. Nat. Rev. Cancer *20*, 743–756. https://doi.org/10.1038/s41568-020-00302-4.

7. Shaffer, S.M., Dunagin, M.C., Torborg, S.R., Torre, E.A., Emert, B., Krepler, C., Beqiri, M., Sproesser, K., Brafford, P.A., Xiao, M., et al. (2017). Rare cell variability and drug-induced reprogramming as a mode of cancer drug resistance. Nature *546*, 431–435. https://doi.org/10.1038/nature22794.

8. Dhimolea, E., de Matos Simoes, R., Kansara, D., Al'Khafaji, A., Bouyssou, J., Weng, X., Sharma, S., Raja, J., Awate, P., Shirasaki, R., et al. (2021). An Embryonic Diapause-like Adaptation with Suppressed Myc Activity Enables Tumor Treatment Persistence. Cancer Cell *39*, 240–256.e11. https://doi.org/10.1016/j.ccell.2020.12.002.

9. Herrera-Abreu, M.T., Palafox, M., Asghar, U., Rivas, M.A., Cutts, R.J., Garcia-Murillas, I., Pearson, A., Guzman, M., Rodriguez, O., Grueso, J., et al. (2016). Early Adaptation and Acquired Resistance to CDK4/6 Inhibition in Estrogen Receptor-Positive Breast Cancer. Cancer Res. *76*, 2301–2313. https://doi.org/10.1158/0008-5472.CAN-15-0728.

10. Yang, C., Li, Z., Bhatt, T., Dickler, M., Giri, D., Scaltriti, M., Baselga, J., Rosen, N., and Chandarlapaty, S. (2017). Acquired CDK6 amplification promotes breast cancer resistance to CDK4/6 inhibitors and loss of ER signaling and dependence. Oncogene *36*, 2255–2264. https://doi.org/10.1038/onc.2016.379.

11. O'Hara, M.H., Jegede, O., Dickson, M.A., DeMichele, A.M., Piekarz, R., Gray, R.J., Wang, V., McShane, L.M., Rubinstein, L.V., Patton, D.R., et al. (2025). Phase II Study of Palbociclib in Patients with Tumors with CDK4 or CDK6 Amplification: Results from the NCI-MATCH ECOG-ACRIN Trial (EAY131) Subprotocol Z1C. Clin. Cancer Res. *31*, 56–64. https://doi.org/10.1158/1078-0432.CCR-24-0036.

12. Li, Z., Razavi, P., Li, Q., Toy, W., Liu, B., Ping, C., Hsieh, W., Sanchez-Vega, F., Brown, D.N., Da Cruz Paula, A.F., et al. (2018). Loss of the FAT1 Tumor Suppressor Promotes Resistance to CDK4/6 Inhibitors via the Hippo Pathway. Cancer Cell *34*, 893–905.e8. https://doi.org/10.1016/j.ccell.2018.11.006.

13. Cornell, L., Wander, S.A., Visal, T., Wagle, N., and Shapiro, G.I. (2019). MicroRNA-Mediated Suppression of the TGF-β Pathway Confers Transmissible and Reversible CDK4/6 Inhibitor Resistance. Cell Rep. *26*, 2667–2680.e7. https://doi.org/10.1016/j.celrep.2019.02.023.

14. Al-Qasem, A.J., Alves, C.L., Ehmsen, S., Tuttolomondo, M., Terp, M.G., Johansen, L.E., Vever, H., Hoeg, L.V.A., Elias, D., Bak, M., and Ditzel, H.J. (2022). Co-targeting CDK2 and CDK4/6 overcomes resistance to aromatase and CDK4/6 inhibitors in ER+ breast cancer. npj Precis. Oncol. *6*, 68. https://doi.org/10.1038/s41698-022-00311-6.

15. Guarducci, C., Bonechi, M., Benelli, M., Biagioni, C., Boccalini, G., Romagnoli, D., Verardo, R., Schiff, R., Osborne, C.K., De Angelis, C., et al. (2018). Cyclin E1 and Rb modulation as common events at time of resistance to palbociclib in hormone receptor-positive breast cancer. NPJ Breast Cancer *4*, 38. https://doi.org/10.1038/s41523-018-0092-4.

16. O'Brien, N.A., McDermott, M.S.J., Conklin, D., Luo, T., Ayala, R., Salgar, S., Chau, K., DiTomaso, E., Babbar, N., Su, F., et al. (2020). Targeting activated PI3K/mTOR signaling overcomes acquired resistance to CDK4/6-based therapies in preclinical models of hormone receptor-positive breast cancer. Breast Cancer Res. *22*, 89. https://doi.org/10.1186/s13058-020-01320-8.

17. Booth, L., West, C., Moore, R.P., Von Hoff, D., and Dent, P. (2022). GZ17-6.02 and palbociclib interact to kill ER+ breast cancer cells. Oncotarget *13*, 92–104. https://doi.org/10.18632/oncotarget.28177.

18. Kim, S., Armand, J., Safonov, A., Zhang, M., Soni, R.K., Schwartz, G., McGuinness, J.E., Hibshoosh, H., Razavi, P., Kim, M., et al. (2023). Sequential activation of E2F via Rb degradation and c-Myc drives resistance to CDK4/6 inhibitors in breast cancer. Cell Rep. *42*, 113198. https://doi.org/10.1016/j.celrep.2023.113198.

19. Kim, S., Son, E., Park, H.-R., Kim, M., and Yang, H.W. (2025). Dual targeting of CDK4/6 and CDK7 augments tumor response and antitumor immunity in breast cancer models. J. Clin. Investig. *135*, e188839. https://doi.org/10.1172/JCI188839.

20. Guarducci, C., Nardone, A., Russo, D., Nagy, Z., Heraud, C., Grinshpun, A., Zhang, Q., Freelander, A., Leventhal, M.J., Feit, A., et al. (2024). Selective CDK7 Inhibition Suppresses Cell Cycle Progression and MYC Signaling While Enhancing Apoptosis in Therapy-resistant Estrogen Receptor–positive Breast Cancer. Clin. Cancer Res. *30*, 1889–1905. https://doi.org/10.1158/1078-0432.CCR-23-2975.

21. Soosainathan, A., Iravani, M., El-Botty, R., Alexander, J., Sourd, L., Morisset, L., Painsec, P., Orha, R., Nikitorowicz-Buniak, J., Pancholi, S., et al. (2024). Targeting Transcriptional Regulation with a CDK9 Inhibitor Suppresses Growth of Endocrine- and Palbociclib-Resistant ER+ Breast Cancers. Cancer Res. *84*, 17–25. https://doi.org/10.1158/0008-5472.CAN-23-0650.

22. Wang, B., Han, J., Elisseeff, J.H., and Demaria, M. (2024). The senescence-associated secretory phenotype and its physiological and pathological implications. Nat. Rev. Mol. Cell Biol. *25*, 958–978. https://doi.org/10.1038/s41580-024-00727-x.

23. Luyties, O., and Taatjes, D.J. (2022). The Mediator kinase module: an interface between cell signaling and transcription. Trends Biochem. Sci. *47*, 314–327. https://doi.org/10.1016/j.tibs.2022.01.002.

24. Chen, M., Li, J., Zhang, L., Wang, L., Cheng, C., Ji, H., Altilia, S., Ding, X., Cai, G., Altomare, D., et al. (2023). CDK8 and CDK19: positive regulators of signal-induced transcription and negative regulators of Mediator complex proteins. Nucleic Acids Res. *51*, 7288–7313. https://doi.org/10.1093/nar/gkad538.

25. Chen, M., Liang, J., Ji, H., Yang, Z., Altilia, S., Hu, B., Schronce, A., McDermott, M.S.J., Schools, G.P., Lim, C.U., et al. (2017). CDK8/19 Mediator kinases potentiate induction of transcription by NFκB. Proc. Natl. Acad. Sci. USA *114*, 10208–10213. https://doi.org/10.1073/pnas.1710467114.

26. McDermott, M.S.J., Chumanevich, A.A., Lim, C.U., Liang, J., Chen, M., Altilia, S., Oliver, D., Rae, J.M., Shtutman, M., Kiaris, H., et al. (2017). Inhibition of CDK8 mediator kinase suppresses estrogen dependent transcription and the growth of estrogen receptor positive breast cancer. Oncotarget *8*, 12558–12575. https://doi.org/10.18632/oncotarget.14894.

27. Donner, A.J., Ebmeier, C.C., Taatjes, D.J., and Espinosa, J.M. (2010). CDK8 is a positive regulator of transcriptional elongation within the serum response network. Nat. Struct. Mol. Biol. *17*, 194–201. https://doi.org/10.1038/nsmb.1752.

28. Galbraith, M.D., Allen, M.A., Bensard, C.L., Wang, X., Schwinn, M.K., Qin, B., Long, H.W., Daniels, D.L., Hahn, W.C., Dowell, R.D., and Espinosa, J.M. (2013). HIF1A employs CDK8-Mediator to stimulate RNAPII elongation. Cell *153*, 1327–1339. https://doi.org/10.1016/j.cell.2013.04.048.

29. Porter, D.C., Farmaki, E., Altilia, S., Schools, G.P., West, D.K., Chen, M., Chang, B.D., Puzyrev, A.T., Lim, C.u., Rokow-Kittell, R., et al. (2012). Cyclin-dependent kinase 8 mediates chemotherapy-induced tumor-promoting paracrine activities. Proc. Natl. Acad. Sci. USA *109*, 13799–13804. https://doi.org/10.1073/pnas.1206906109.

30. Sharko, A.C., Lim, C.U., McDermott, M.S.J., Hennes, C., Philavong, K.P., Aiken, T., Tatarskiy, V.V., Roninson, I.B., and Broude, E.V. (2021). The Inhibition of CDK8/19 Mediator Kinases Prevents the Development of Resistance to EGFR-Targeting Drugs. Cells *10*, 144. https://doi.org/10.3390/cells10010144.

31. Chen, B., Wen, P., Hu, G., Gao, Y., Qi, X., Zhu, K., Chen, S., Wu, L., Xu, A., and Zhao, G. (2020). Antagonizing CDK8 Sensitizes Colorectal Cancer to Radiation Through Potentiating the Transcription of e2f1 Target Gene apaf1. Front. Cell Dev. Biol. *8*, 408. https://doi.org/10.3389/fcell.2020.00408.

32. Malone, C.F., Kim, M., Alexe, G., Engel, K., Forman, A.B., Robichaud, A., Conway, A.S., Goodale, A., Meyer, A., Khalid, D., et al. (2023).

CellPress
OPEN ACCESS

Transcriptional Antagonism by CDK8 Inhibition Improves Therapeutic Efficacy of MEK Inhibitors. Cancer Res. *83*, 285–300. https://doi.org/10.1158/0008-5472.CAN-21-4309.

33. Ding, X., Sharko, A.C., McDermott, M.S.J., Schools, G.P., Chumanevich, A., Ji, H., Li, J., Zhang, L., Mack, Z.T., Sikirzhytski, V., et al. (2022). Inhibition of CDK8/19 Mediator kinase potentiates HER2-targeting drugs and bypasses resistance to these agents in vitro and in vivo. Proc. Natl. Acad. Sci. *119*, e2201073119. https://doi.org/10.1073/pnas.2201073119.

34. Ding, X., Liang, J., Sharko, A.C., Hilimire, T.A., Li, J., Loskutov, J., Mack, Z.T., Ji, H., Schools, G.P., Cai, C., et al. (2024). Mediator kinase inhibitors suppress triple-negative breast cancer growth and extend tumor suppression by mTOR and AKT inhibitors. Proc. Natl. Acad. Sci. *121*, e2414501121. https://doi.org/10.1073/pnas.2414501121.

35. Nussbaum, D.P., Martz, C.A., Waters, A.M., Barrera, A., Liu, A., Rutter, J.C., Cerda-Smith, C.G., Stewart, A.E., Wu, C., Cakir, M., et al. (2024). Mediator kinase inhibition impedes transcriptional plasticity and prevents resistance to ERK/MAPK-targeted therapy in KRAS-mutant cancers. npj Precis. Oncol. *8*, 124. https://doi.org/10.1038/s41698-024-00615-9.

36. Li, J. (2023). CDK19 and CDK8 Mediator Kinases Drive Androgen-Independent in Vivo Growth of Castration-Resistant Prostate Cancer. Preprint at bioRxiv. https://doi.org/10.1101/2023.08.08.552491.

37. Vijayaraghavan, S., Karakas, C., Doostan, I., Chen, X., Bui, T., Yi, M., Raghavendra, A.S., Zhao, Y., Bashour, S.I., Ibrahim, N.K., et al. (2017). CDK4/6 and autophagy inhibitors synergistically induce senescence in Rb positive cytoplasmic cyclin E negative cancers. Nat. Commun. *8*, 15916. https://doi.org/10.1038/ncomms15916.

38. Zhang, L., Cheng, C., Li, J., Wang, L., Chumanevich, A.A., Porter, D.C., Mindich, A., Gorbunova, S., Roninson, I.B., Chen, M., and McInnes, C. (2022). A Selective and Orally Bioavailable Quinoline-6-Carbonitrile-Based Inhibitor of CDK8/19 Mediator Kinase with Tumor-Enriched Pharmacokinetics. J. Med. Chem. *65*, 3420–3433. https://doi.org/10.1021/acs.jmedchem.1c01951.

39. Hofmann, M.H., Mani, R., Engelhardt, H., Impagnatiello, M.A., Carotta, S., Kerenyi, M., Lorenzo-Herrero, S., Böttcher, J., Scharn, D., Arnhof, H., et al. (2020). Selective and Potent CDK8/19 Inhibitors Enhance NK-Cell Activity and Promote Tumor Surveillance. Mol. Cancer Ther. *19*, 1018–1030. https://doi.org/10.1158/1535-7163.MCT-19-0789.

40. Grandjean, J.-M.M., Jiu, A.Y., West, J.W., Aoyagi, A., Droege, D.G., Elepano, M., Hirasawa, M., Hirouchi, M., Murakami, R., Lee, J., et al. (2020). Discovery of 4-Piperazine Isoquinoline Derivatives as Potent and Brain-Permeable Tau Prion Inhibitors with CDK8 Activity. ACS Med. Chem. Lett. *11*, 127–132. https://doi.org/10.1021/acsmedchemlett.9b00480.

41. Comşa, Ş., Cîmpean, A.M., and Raica, M. (2015). The Story of MCF-7 Breast Cancer Cell Line: 40 years of Experience in Research. Anticancer Res. *35*, 3147–3154.

42. Vargas-Rondón, N., Pérez-Mora, E., Villegas, V.E., and Rondón-Lagos, M. (2020). Role of chromosomal instability and clonal heterogeneity in the therapy response of breast cancer cell lines. Cancer Biol. Med. *17*, 970.

43. Luria, S.E., and Delbrück, M. (1943). Mutations of Bacteria from Virus Sensitivity to Virus Resistance. Genetics *28*, 491–511. https://doi.org/10.1093/genetics/28.6.491.

44. Chang, C.A., Jen, J., Jiang, S., Sayad, A., Mer, A.S., Brown, K.R., Nixon, A.M.L., Dhabaria, A., Tang, K.H., Venet, D., et al. (2022). Ontogeny and Vulnerabilities of Drug-Tolerant Persisters in HER2+ Breast Cancer. Cancer Discov. *12*, 1022–1045. https://doi.org/10.1158/2159-8290.CD-20-1265.

45. Singh, A., and Saint-Antoine, M. (2022). Probing transient memory of cellular states using single-cell lineages. Front. Microbiol. *13*, 1050516. https://doi.org/10.3389/fmicb.2022.1050516.

46. Serrao, A., Jenkins, L.M., Chumanevich, A.A., Horst, B., Liang, J., Gatza, M.L., Lee, N.Y., Roninson, I.B., Broude, E.V., and Mythreye, K. (2018). Mediator kinase CDK8/CDK19 drives YAP1-dependent BMP4-induced EMT in cancer. Oncogene *37*, 4792–4808. https://doi.org/10.1038/s41388-018-0316-y.

47. Liang, J., Chen, M., Hughes, D., Chumanevich, A.A., Altilia, S., Kaza, V., Lim, C.U., Kiaris, H., Mythreye, K., Pena, M.M., et al. (2018). CDK8 selectively promotes the growth of colon cancer metastases in the liver by regulating gene expression of TIMP3 and matrix metalloproteinases. Cancer Res. *78*, 6594–6606. https://doi.org/10.1158/0008-5472.CAN-18-1583.

48. Chen, H., Li, Y., and Tollefsbol, T.O. (2013). Cell Senescence Culturing Methods. Methods Mol. Biol. *1048*, 1–10. https://doi.org/10.1007/978-1-62703-556–9_1.

49. Song, F., Zhang, Q., Lu, X., Xu, T., Hu, Q., Hu, X., Fan, W., Zhang, Y., and Huang, P. (2023). Rab11-FIP4 interacts with ARF5 to promote cancer stemness in hepatocellular carcinoma. J. Physiol. Biochem. *79*, 757–770. https://doi.org/10.1007/s13105-023-00972-2.

50. Novototskaya-Vlasova, K.A., Neznanov, N.S., Molodtsov, I., Hall, B.M., Commane, M., Gleiberman, A.S., Murray, J., Haber, M., Norris, M.D., Leonova, K.I., and Gudkov, A.V. (2022). Inflammatory response to retrotransposons drives tumor drug resistance that can be prevented by reverse transcriptase inhibitors. Proc. Natl. Acad. Sci. *119*, e2213146119. https://doi.org/10.1073/pnas.2213146119.

51. Chen, F., Ding, K., Priedigkeit, N., Elangovan, A., Levine, K.M., Carleton, N., Savariau, L., Atkinson, J.M., Oesterreich, S., and Lee, A.V. (2021). Single-cell transcriptomic heterogeneity in invasive ductal and lobular breast cancer cells. Cancer Res. *81*, 268–281. https://doi.org/10.1158/0008-5472.CAN-20-0696.

52. Brown, T.C., Murtha, T.D., Rubinstein, J.C., Korah, R., and Carling, T. (2018). SLC12A7 alters adrenocortical carcinoma cell adhesion properties to promote an aggressive invasive behavior. Cell Commun. Signal. *16*, 27. https://doi.org/10.1186/s12964-018-0243-0.

53. Li, D., San, M., Zhang, J., Yang, A., Xie, W., Chen, Y., Lu, X., Zhang, Y., Zhao, M., Feng, X., and Zheng, Y. (2021). Oxytocin receptor induces mammary tumorigenesis through prolactin/p-STAT5 pathway. Cell Death Dis. *12*, 588. https://doi.org/10.1038/s41419-021-03849-8.

54. Angel, C.Z., Beattie, S., Hanif, E.A.M., Ryan, M.P., Guerra Liberal, F.D.C., Zhang, S.D., Monteith, S., Buckley, N.E., Parker, E., Haynes, S., et al. (2024). A SRC-slug-TGFβ2 signaling axis drives poor outcomes in triple-negative breast cancers. Cell Commun. Signal. *22*, 454. https://doi.org/10.1186/s12964-024-01793-6.

55. Price, M.J., Nguyen, A.D., Byemerwa, J.K., Flowers, J., Baëta, C.D., and Goodwin, C.R. (2023). UDP-glucose dehydrogenase (UGDH) in clinical oncology and cancer biology. Oncotarget *14*, 843–857. https://doi.org/10.18632/oncotarget.28514.

56. Reuveni, H., Flashner-Abramson, E., Steiner, L., Makedonski, K., Song, R., Shir, A., Herlyn, M., Bar-Eli, M., and Levitzki, A. (2013). Therapeutic Destruction of Insulin Receptor Substrates for Cancer Treatment. Cancer Res. *73*, 4383–4394. https://doi.org/10.1158/0008-5472.CAN-12-3385.

57. Singh, P., Alex, J.M., and Bast, F. (2014). Insulin receptor (IR) and insulin-like growth factor receptor 1 (IGF-1R) signaling systems: novel treatment strategies for cancer. Med. Oncol. *31*, 805. https://doi.org/10.1007/s12032-013-0805-3.

58. Wang, D., Chen, K., Wang, Z., Wu, H., and Li, Y. (2024). Research progress on interferon and cellular senescence. FASEB J. *38*, e70000. https://doi.org/10.1096/fj.202400808RR.

59. Bancerek, J., Poss, Z.C., Steinparzer, I., Sedlyarov, V., Pfaffenwimmer, T., Mikulic, I., Dölken, L., Strobl, B., Müller, M., Taatjes, D.J., and Kovarik, P. (2013). CDK8 kinase phosphorylates transcription factor STAT1 to selectively regulate the interferon response. Immunity *38*, 250–262. https://doi.org/10.1016/j.immuni.2012.10.017.

60. Yamamoto, S., Hagihara, T., Horiuchi, Y., Okui, A., Wani, S., Yoshida, T., Inoue, T., Tanaka, A., Ito, T., Hirose, Y., and Ohkuma, Y. (2017). Mediator cyclin-dependent kinases upregulate transcription of inflammatory genes in cooperation with NF-κB and C/EBPβ on stimulation of Toll-like receptor 9. Genes Cells *22*, 265–276. https://doi.org/10.1111/gtc.12475.

61. Wei, R., Kong, L., Xiao, Y., Yuan, H., Song, Y., Wang, J., Yu, H., Mao, S., and Xu, W. (2018). CDK8 regulates the angiogenesis of pancreatic cancer cells in part via the CDK8-β-catenin-KLF2 signal axis. Exp. Cell Res. *369*, 304–315. https://doi.org/10.1016/j.yexcr.2018.05.033.
62. Dobin, A., Davis, C.A., Schlesinger, F., Drenkow, J., Zaleski, C., Jha, S., Batut, P., Chaisson, M., and Gingeras, T.R. (2013). STAR: ultrafast universal RNA-seq aligner. Bioinformatics *29*, 15–21. https://doi.org/10.1093/bioinformatics/bts635.
63. Love, M.I., Huber, W., and Anders, S. (2014). Moderated estimation of fold change and dispersion for RNA-seq data with DESeq2. Genome Biol. *15*, 550. https://doi.org/10.1186/s13059-014-0550-8.
64. Korotkevich, G., Sukhov, V., Budin, N., Shpak, B., Artyomov, M.N., and Sergushichev, A. (2021). Fast gene set enrichment analysis. Preprint at bioRxiv. https://doi.org/10.1101/060012.
65. Wickham, H., Averick, M., Bryan, J., Chang, W., McGowan, L., François, R., Grolemund, G., Hayes, A., Henry, L., Hester, J., et al. (2019). Welcome to the Tidyverse. J. Open Source Softw. *4*, 1686. https://doi.org/10.21105/joss.01686.
66. Kolde, R. pheatmap: Pretty Heatmaps. 1.0.13 https://doi.org/10.32614/CRAN.package.pheatmap (2010).

STAR★METHODS

KEY RESOURCES TABLE

REAGENT or RESOURCE	SOURCE	IDENTIFIER
**Antibodies**		
Rb (4H1)	Cell Signaling Technology	Cat# 9309, RRID:AB_823629
Phospho-Rb (Ser780) (D59B7) Rabbit mAb	Cell Signaling Technology	Cat# 8180, RRID:AB_10950972
Phospho-Rb (Ser807/811) (D20B12) XP Rabbit mAb	Cell Signaling Technology	Cat# 8516, RRID:AB_11178658
E2F1(KH95)	Thermo Fisher Scientific	Cat# 32–1400, RRID: AB_2533065
Cyclin E1 (HE12)	Cell Signaling Technology	Cat# 4129, RRID:AB_2071200
β-Actin	Sigma-Aldrich	Cat# A2228, RRID:AB_476697
GAPDH	Cell Signaling Technology	Cat# 2118, RRID:AB_561053
Ki-67 (D2H10)	Cell Signaling Technology	Cat# 9027, RRID:AB_2636984
Donkey anti-Rabbit IgG Secondary Antibody, Alexa Fluor™ Plus 555	Thermo Fisher Scientific	Cat# A32794, RRID:AB_2762834
Anti-rabbit IgG, HRP-linked Antibody	Cell Signaling Technology	Cat# 7074, RRID:AB_2099233
Anti-mouse IgG, HRP-linked Antibody	Cell Signaling Technology	Cat# 7076, RRID:AB_330924
**Bacterial and virus strains**		
NEB® Stable Competent E. coli (High Efficiency)	New England Biolabs	Cat# C3040I
**Chemicals, peptides, and recombinant proteins**		
Senexin B	Senex Biotechnology	N/A
SNX631	Senex Biotechnology	N/A
SNX631-6	Senex Biotechnology	N/A
Puromycin dihydrochloride	Tocris Bioscience	Cat# 408950
Blasticidin S, Hydrochloride	MilliporeSigma™	Cat# 20335025MG
Halt Protease Inhibitor Cocktail	Thermo Scientific™	Cat# 78438
D-MEM (high-glucose)	Cytiva	Cat# SH30022.01
RPMI-1640	Cytiva/Hyclone	Cat# SH30027.01
Fetal Bovine Serum (FBS)	Cytiva/Hyclone	Cat# SH30396.03
Sodium Pyruvate, 100 mM	Cytiva/Hyclone	Cat# SH30239.01
Penicillin/Streptomycin/Glutamine (100x)	Cytiva/Hyclone	Cat# SV30010
Corning™ Matrigel™ Membrane Matrix	Fisher Scientific	Cat# CB-40234
**Critical commercial assays**		
MycoAlert™ Mycoplasma Detection Kit	Lonza	Cat# LT07
DC Protein Assay Kit	Bio-Rad Laboratories	Cat# 5000116
RNeasy Mini Kit	QIAGEN	Cat# 74106
RNAlater stabilization solution	Thermo Fisher Scientific	Cat# AM7020
miRNeasy Mini Kit	QIAGEN	Cat# 74106
NEBNext Ultra II Directional RNA Library Prep Kit	New England Biolabs	Cat# E7760
Plasmid mini prep Kit	QIAGEN	Cat# 2710
QiaQuick Gel Extraction Kit	QIAGEN	Cat# 28706
CloneDirect™ Rapid Ligation Kit	Lucigen	Cat# 40020
Western Lightning® Plus-ECL reagent	PerkinElmer Inc.	Cat# NEL105001EA
**Deposited data**		
RNA-Seq raw data	NCBI Gene Expression Omnibus (GEO)	GSE303026

(*Continued on next page*)

Continued		
REAGENT or RESOURCE	SOURCE	IDENTIFIER
Experimental models: Cell lines		
HEK293 (293)	ATCC	CRL-1573; RRID: CVCL_0045
MCF-7	ATCC	CRL-12584; RRID: CVCL_0031
T-47D	ATCC	HTB-133; RRID: CVCL_0553
SW-620	ATCC	CCL-227; RRID: CVCL_0547
ZR-75-1	ATCC	CRL-1500; RRID: CVCL_0588
Experimental models: Organisms/strains		
NOD.Cg-Prkdc^{scid} Il2rg^{tm1Wjl}/SzJ Mice	The Jackson laboratory and Dr. Marj Pena, U of SC	IMSR_JAX:005557
Oligonucleotides		
GAACTTAGTAGCAAAAGACCCAGA	This paper	N/A
GATAAAGATACAGATTCCCCACAG	This paper	N/A
Software and algorithms		
GraphPad Prism 10	Graph Pad Software	RRID:SCR_002798
Image Lab version 5.2.1 build 11	Bio-Rad	RRID:SCR_014210
Bio-Rad CFX Manager 3.0	Bio-Rad	Bio-Rad CFX Manager 3.0
STAR version 2.7.2b	Dobin et al.[62]	RRID:SCR_004463
R version 4.5.0	R Core Team	https://www.R-project.org/
DeSeq2 package	Love et al.[63]	RRID:SCR_015687
fgsea	Korotkevich et al.[64]	RRID:SCR_020938
tidyverse	Wickham et al.[65]	RRID:SCR_019186
pheatmap	Kolde R.[66]	RRID:SCR_016418

EXPERIMENTAL MODEL AND STUDY PARTICIPANT DETAILS

Cell lines

All cell lines were obtained from ATCC and grown in an incubator at 37°C and 5% CO2, and all base medium was supplemented with 10% fetal bovine serum (FBS; Cytiva, Marlborough, MA), 1% penicillin/streptomycin, and 2 mM L-glutamine. MCF-7 parental cell line and its clones were maintained in DMEM-high glucose media (ThermoFisher Scientific, Waltham, MA) supplemented with 1 mM sodium pyruvate (HyClone, Logan, Utah), and 0.01 mg/mL human recombinant insulin (ThermoFisher Scientific, Waltham, MA). ZR-75-1 cells were maintained in DMEM-high glucose media. T-47D cells were maintained in RPMI-1640 media (HyClone, Logan, Utah). SW-620 cells were maintained in Leibowitz's L-15 base media (HyClone, Logan, Utah). All cells used for experiments were confirmed mycoplasma-free by MycoAlert Plus mycoplasma detection kit (Lonza, Morrisville, NC).

Mice

All mouse studies were approved by the University of South Carolina Institutional Animal Care and Use Committee protocol 2688-101856-103023. Female NOD.Cg-Prkdc^{scid} Il2rg^{tm1Wjl}/SzJ (NSG) mice (aged 6 weeks) were obtained from The Jackson Laboratory (Bar Harbor, ME) or from the lab of Marj Pena at the University of South Carolina (USC). Jackson Laboratory mice were allowed to habituate to the USC Animal Research Facility for 7–10 days. Mice were housed in solid bottom polycarbonate ventilated cages at a temperature of 68–79°F in 30–70% humidity. Mice were kept on a 12-h light, 12-h dark cycle with regularly changed 100% filtered air. To support growth of estrogen receptor-positive tumor cells, mice were given sterile H_2O supplemented with 8 μg/mL β-estradiol for 1 week prior to inoculation, which was subsequently refreshed weekly throughout the duration of the study. For the MCF-7 study, 5×10^6 cells were suspended in 50% Matrigel (Corning, Oneonta, NY)/50% sterile phosphate-buffered saline) and injected subcutaneously into the right flank. For the T-47D study, 2.5×10^6 cells were suspended in 50% Matrigel (Corning, Oneonta, NY)/50% sterile phosphate-buffered saline) and injected subcutaneously into the right flank. Tumors were measured twice weekly using calipers and tumor volume was calculated as 0.5 × length (mm) × width (mm)^2. Once average tumor volume reached ~150mm^3, mice were randomized to 4 treatment groups: control, SNX631-6 alone, palbociclib alone, or SNX631-6 and palbociclib combination. 50 mg/kg palbociclib isethionate in H_2O was administered p.o. daily for 5 days, followed by a 2-day drug holiday. For the MCF-7 study, 30 mg/kg SNX631-6 in 70% PEG400/30% PG was administered p.o. q.d. For the T-47D study, SNX631-6 was administered by 350 ppm medicated diet, *ad libitum* (Research Diets, New Brunswick, NJ). On the final day of the experiment or at tumor size

endpoint, tumors were excised, weighed, and divided into pieces to be fixed in RNA Later or 10% formalin for 24 h and stored in 70% ethanol at 4°C until processing.

METHOD DETAILS

SNX631, SNX631-6, Senexin C, and Senexin B were synthesized for Senex Biotechnology (Columbia, SC); BI-1347 and Compound 25 was synthesized by Drug Design and Synthesis Core of the USC COBRE Center for Targeted Therapeutics. Palbociclib isethionate was purchased from SelleckChem (#S1116, Houston, TX). Abemaciclib (KP-736-002) was provided by Kalyra Pharmaceuticals (Batch MW: 506.59 g/mol; Purity: 97%). Ribociclib succinate (HY-15777B) was purchased from MedChemExpress.

Cell proliferation assays

Cells were seeded into 96-well plates (1,500 cells/well). After 24 h, cells were treated with palbociclib (4 nM-1 μM or 16 nM-4 μM), alone or in combination with SNX631 (500 nM). After 7 days, cell densities were measured by sulforhodamine B sodium salt (SRB) assay. For 20-day SRB time course assays, culture media were replaced every 5 days. For flask assays, cells were seeded into T25 flasks (250,000 cells/flask for the lower-dose SNX631 study; 600,000 cells/flask for the higher-dose Senexin B study). Vehicle control- and 500 nM SNX631-treated flasks were stained with crystal violet when the vehicle control flask reached 100% confluence. 500 nM palbociclib- and 500 nM palbociclib+SNX631-treated flasks were stained with crystal violet when the combination flask was estimated to contain no viable cells. Crystal violet images are representative of three separate biological replicates. For 20-day raw cell counting experiments, cells were seeded into duplicate flasks per condition (125,000 cells per flask) and treated with the appropriate drug conditions the day after seeding. Culture media were replaced every 7 days with fresh, drug-containing media. At each indicated time point, duplicate flasks were trypsinized, and total live cell numbers were counted by Bio-Rad TC10 automated cell counter with trypan blue dye. For 25-day endpoint analysis, triplicate flasks per condition were trypsinized and counted, and a fourth replicate flask was stained with crystal violet.

Bliss synergy analysis

Bliss synergy analysis was performed to assess the interaction between Palbociclib and SNX631 in MCF-7 cells. Cells were treated with each drug alone and in combination at the indicated concentrations, and cell viability was measured following the specified treatment period and normalized to untreated controls. The expected combined effect under the Bliss independence model (E_bliss) was calculated as $E\_bliss = E\_A + E\_B - (E\_A \times E\_B)$, where E_A and E_B represent the fractional effects of Palbociclib and SNX631, respectively. Bliss scores were determined by comparing the observed combination effect to the expected effect, with positive values indicating synergy, negative values indicating antagonism, and values near zero indicating additive effects.

Senescence-associated-B-galactosidase staining

10^4 cells per cm^2 of vessel were seeded, incubated for 12-24 h to allow for attachment, and treated with DMSO, SNX631 (500 nM), palbociclib (500 nM), or 500 nM SNX631 and 500 nM palbociclib combination. Post-treatment, cells were washed 3 times with phosphate-buffered saline (PBS) (pH = 7.4) before being covered in a fixation solution (2% Formaldehyde and 0.2% Glutaraldehyde solution in PBS, made freshly each experiment) for 5–10 min and left at room temperature. After aspiration of the fixative and subsequent 3 washes with PBS, a staining solution (made in 10 mL aliquots using 6.3 mL dH_2O, 0.8 mL 0.5 M Citric Acid/Na_2HPO_4 [pH 6.0], 1.0 mL 50 mM $K_3Fe(CN)_6$, 1.0 mL 50 mM $K_4Fe(CN)_6$, 0.3 mL 5 M NaCl, 0.02 mL 1 M $MgCl_2$-$6H_2O$, and 0.5 mL 20 mg/mL X-gal [GOLD Biotechnology, Inc., Olivette, MO]) was added to cover the cells and incubated at 37°C for 24–48 h. After the final 3 washes with PBS, nuclei were counterstained with a solution of 1μM 4′,6-diamidino-2-phenylindole (DAPI) as cells were covered with a 50% glycerol solution in H_2O and stored at 4°C.

Cell cloning

One single MCF-7 cell per well was plated into ten 96-well plates. Colonies were allowed to proliferate for a week before being observed daily using Agilent BioTek Cytation V Live Cell Imaging System. Once exponential growth was achieved from a single cell to create a colony, cells were trypsinized, expanded, and eventually frozen in 10% DMSO/90% media.

Time-lapse video microscopy

10^3 MCF-7 cells per well in a 6-well plate were seeded, incubated for 12-24 h to allow for attachment, and treated with DMSO, SNX631 (500 nM), palbociclib (500 nM), or 500nM SNX631 and 500 nM palbociclib combination. Post-treatment, each well was comprehensively imaged using Agilent BioTek Cytation V Live Cell Imaging System every 6 h over 26 days.

RNA-SEQ

MCF-7 cells were treated with DMSO, SNX631 (500 nM), palbociclib (500 nM), or a combination of 500 nM SNX631 and 500 nM palbociclib. Two distinct protocols were employed for a 3-day and a 28-day duration. Library preparation, raw data post-processing, and data analysis were performed by the USC CTT COBRE Functional Genomics Core. In the 3-day experiment, 3′-tag-seq RNA libraries were prepared using the QuantSeq 3′ mRNA-Seq Library Prep Kit FWD for Illumina, with a starting material of 1000 ng

per sample. Illumina NextSeq500 (75bp, pair-ended) was utilized for sequencing, and reads were aligned to the Human genome GRCh38.p13 using STAR v2.7.2b99. The average read depth was 2 million reads, and gene expression analysis utilized the edgeR package with normalization by the Trimmed Mean of M-values (TMM) method. Only uniquely mapped reads were considered, and differential expression analysis included correction for multiple testing using Benjamini-Hochburg's FDR. For the 28-day experiment, RNA libraries were prepared with the NEBNext poly(a) mRNA magnetic isolation kit, Ultra II Directional Library Prep Kit, and SIRV-Set 3 (Lexogen Spike-in kit) using 500 ng starting material per sample. Illumina HiSeqX (150bp, pair-ended) by Medgenome was employed for sequencing, and the analysis protocol mirrored that of the 3-day experiment, with the primary distinctions being the increased starting material (500 ng) and a substantially higher average read depth of 79 million reads. Differential expression (DE) analysis was performed in R using the DESeq2 (30) pipeline, where the normalized counts data were fit to a negative binomial distribution model using a generalized linear model (GLM) framework and the Benjamini-Hochberg procedure was used to control the false discovery rate (FDR) for multiple testing. The logFC and FDR values calculated from DESeq2 pipelines were utilized to select differentially expressed genes (DEGs). To select validated DEGs from multiple RNA-Seq experiments, the following criteria were applied: (1) FDR <0.05 in all experiments; (2) average log2FC from multiple experiments > log2(1.5). The gene set enrichment analysis (GSEA) for different comparisons was conducted using the fgsea package with the specific gene sets downloaded from the Human Molecular Signatures Database (MSigDB). All raw and processed RNA-Seq data have been uploaded to GEO (see data availability section).

Lentiviral knockdown of RB

Gene-specific shRNA sequences targeting human Rb were cloned into the pLKO.1-puro lentiviral construct (Addgene #8453). The oligonucleotides used for cloning were ordered from IDT: GAACTTAGTAGCAAAAGACCCAGA targeting the 3′UTR (Rb-shRNA#1) and GATAAAGATACAGATTCCCCACAG targeting the coding sequence (CDS) (Rb-shRNA#2). A scrambled shRNA control was purchased from Sigma. Lentivirus was packaged by co-transfecting HEK293T cells with 1.5 μg pMD2.G, 4.5 μg psPAX2, and 6 μg shRNA construct. Viral supernatants were collected at 48 and 72 h post-transfection and filtered through a 0.45 μm membrane. Rb-knockdown derivatives were generated by selection with 2 μg/mL puromycin following lentiviral transduction.

Western blot

10^4 cells per vessel cm^2 were seeded, incubated for 12-24 h to allow for attachment, and treated with DMSO, SNX631 (500 nM), palbociclib (500 nM), or 500 nM SNX631 and 500 nM palbociclib combination. Treated and control cells were then lysed using RIPA Lysis Buffer (50 mM Tris-HCl pH 8.0, 150 mM NaCl, 0.1% sodium dodecyl sulfate, 1% NP-40, 2 mM phenylmethylsulfonyl fluoride (VWR Life Science, Radnor, PA) supplemented with SIGMAFASTTM Protease Inhibitor Cocktail (#S8820, Sigma Aldrich, St Louis, MO). Whole cell lysates were resolved on 10% PAAG, transferred to 0.22 μm nitrocellulose membranes (Bio-Rad, Hercules, CA), blocked with 5% non-fat milk, and incubated with primary antibodies diluted 1:1000 in 1% BSA in TBS overnight at 4°C: Rb (4H1) (#9309, 1:1000; Cell Signaling Technology, Danvers, MA), phospho-Rb Ser780 (D59B7) (#8180, 1:1000; Cell Signaling Technology), phospho-Rb Ser807/811 (D20B12) (#8516, 1:1000; Cell Signaling Technology), E2F1(KH95) (#32–1400, 1:1000, Invitrogen, Waltham, MA), Cyclin E1 (HE12) (#4129, 1:1000, Cell Signaling Technology), beta-actin (AC-74) (#A2228, 1:5000, Sigma Aldrich), and GAPDH (#2118, 1:1000; Cell Signaling Technology). Membranes were then washed with TBS-T and blotted with HRP-conjugated anti-Mouse (#7076, Cell Signaling Technology) or anti-Rabbit (#7074, Cell Signaling Technology) secondary antibodies diluted 1:1000 in 5% non-fat milk. Protein bands were detected with Clarity Western ECL Substrate (Bio-Rad) using the iBright FL1500 Imaging System (Invitrogen).

Analysis of publicly available RNA-seq data

NCBI Gene Expression Omnibus (GEO) Series GSE144320 (MCF-7 scRNA-Seq) raw counts files were converted to Reads Per Kilobase per Million (RPKM) and compared to DEGs overexpressed ($Log_2(FC) \geq 2$; fpkm >5) in palbociclib-treated cells from our LT bulk RNA-Seq. Among the 977 individual cells, only those with nonzero RPKM are plotted. The 129 atirmociclib-resistance associated genes were extracted from the the Supplemental information file Table S5 of Palmer et al.[5]

Immunofluorescence (IF) labeling, H&E staining, and analysis

For IF of formalin-fixed and paraffin-embedded tissue blocks, tissues were blocked in 5% normal donkey serum in TBST for 1 h. The following primary antibodies were diluted in 1% BSA in PBST and then incubated on the sections overnight at 4°C: Ki67 (Cell Signaling #9027, 1:2000 dilution). Alexa Fluor 555 labeled secondary antibodies were used at 1:200 for 1 h. Nuclei were counterstained with 1 mM 4′,6-diamidino-2-phenylindole (DAPI) and sections were mounted in Prolong Glass. For IF and H&E analysis, image montages were acquired on Agilent (BioTek, VT, US) Cytation V with a 10×/0.3 n.a. objective, using identical acquisition parameters for all sections. For fluorescence intensity measurements, tumor regions of interest were manually defined using the DAPI channel to select at least three areas of cancer cells in each section. Ki67 mean pixel intensity quantification was measured within these regions of interest using ImageJ. The labeling, imaging, and quantification were repeated two times, with similar results. H&E-stained sections from the MCF-7 *in vivo* study were scored by the following criteria: 0 - No Muscle tissue is present in the image; 1 - Muscle/tumor border is visible, but no invasion is present; 2 - Minimal depth/quantity of invasion; 3 - Moderate depth/quantity of invasion; 4 - Severe invasion/complete integration of tumor and muscle.

CellPress
OPEN ACCESS

QUANTIFICATION AND STATISTICAL ANALYSIS

Independent experiments were conducted with a minimum of two biological replicates per condition to allow for statistical comparison. To evaluate treatment effects in 20-day SRB assays, one-way ANOVA was performed at each time point, restricting the analysis to experimental conditions that had not reached assay saturation (defined as OD 570 nm < 2.5). Post hoc multiple comparisons were conducted using Šidák's multiple comparisons test to control for overall Type I error. The numbers of biologically independent experiments, the number of individual mice and details of statistical tests performed can be found in the respective figure legends, results, or method details sections. Information on data quantification and analysis methods is provided under method details. For statistical analysis, Student's *t* test or ANOVA was used. Statistical significance was indicated with *p* values *$p < 0.05$, **$p < 0.01$, ***$p < 0.001$, ****$p < 0.0001$ in figures.

Cell Reports Medicine, Volume *7*

Supplemental information

CDK8/19 inhibition prevents adaptive resistance to CDK4/6 inhibitors *in vitro* and *in vivo*

Zachary T. Mack, Margarita Yastrebova, Chang-uk Lim, Xiaokai Ding, Jing Li, Alvina Khamidullina, Amanda C. Sharko, Michael Saint-Antoine, Thomas A. Hilimire, Gary P. Schools, Kaitlyn Digsby, Amanda Eckstrom, Erin Belcher, Gwenael Madiot, Hao Ji, Vitali Sikirzhitski, Chao Cai, Guoshuai Cai, Abhyudai Singh, Victor Tatarskiy, Mengqian Chen, Igor B. Roninson, and Eugenia V. Broude

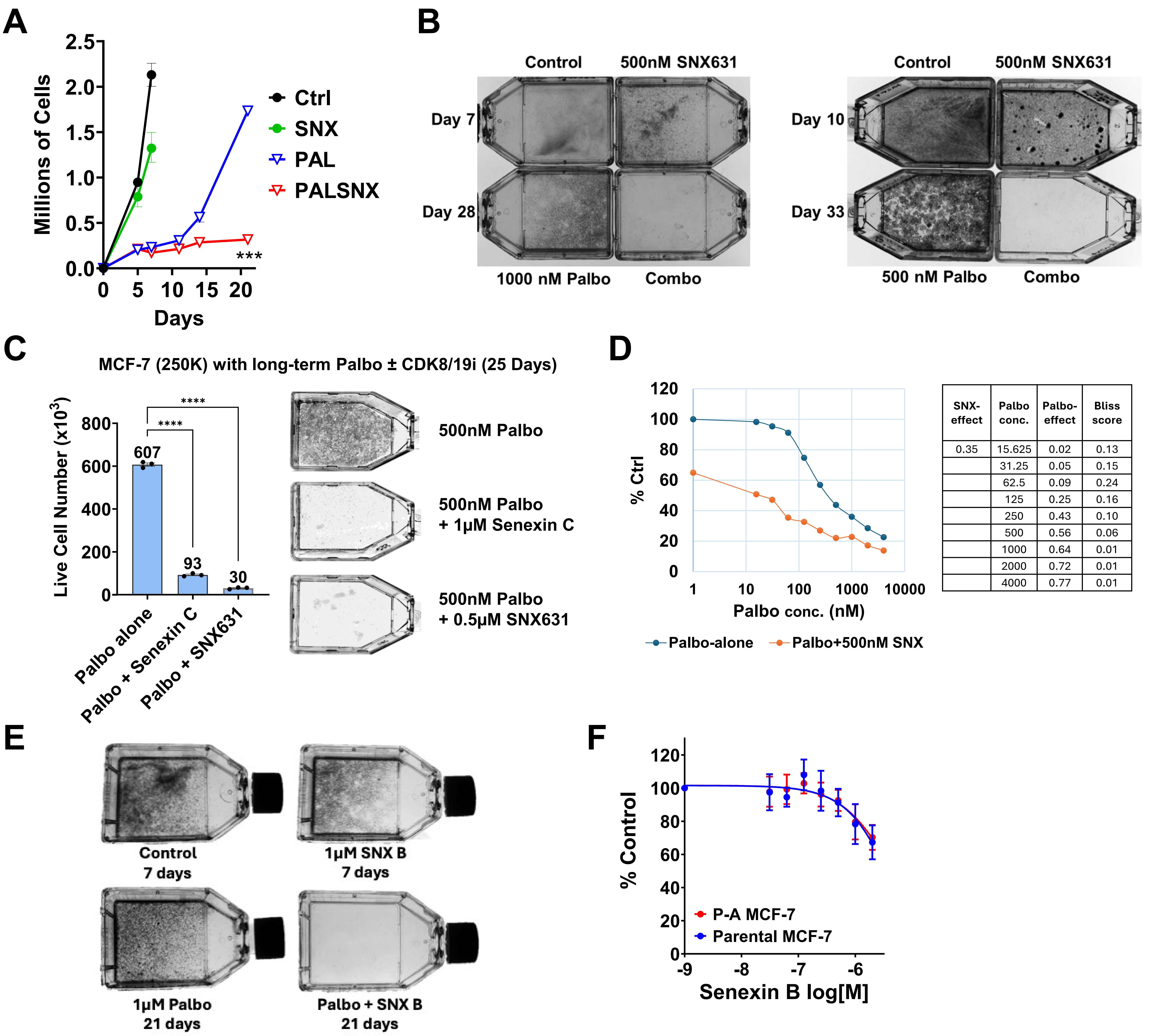

Figure S1. CDK8/19 inhibitors enhance the long-term growth suppression of palbociclib in MCF-7 cells. Related to Figures 1. **(A)** Raw cell counts from 20-day viability analyses of MCF-7 cells treated with 500 nM palbociclib with or without 500 nM SNX631. Statistics: unpaired two-tailed Welch's t-test; ***: P < 0.001. **(B)** Long-term palbociclib-treated (500 or 1000 nM) and combination-treated flasks were stained with crystal violet once apparent palbociclib adaptation was observed in the palbociclib-treated flasks. **(C)** Final cell counts and crystal violet-stained flasks after 25 days of treatment of MCF-7 cells with 500 nM palbociclib alone or with 1 µM Senexin C or 500 nM SNX631. Statistics: ordinary one-way ANOVA with Šídák's multiple comparisons test; ****: P < 0.0001. **(D)** Synergy analysis of different concentrations of palbociclib combined with 500 nM SNX631 in MCF-7 cells. **(E)** Representative images of crystal violet-stained flasks. Untreated and 1 µM Senexin B (SNX B)-treated MCF-7 cells were stained at 7 days, while cells treated with 1 µM palbociclib with and without Senexin B were stained at 21 days. **(F)** Seven-day growth assay of parental and palbociclib-adapted (P-A) MCF-7 cells treated with Senexin B at different concentrations. Data are represented as mean +/- SEM.

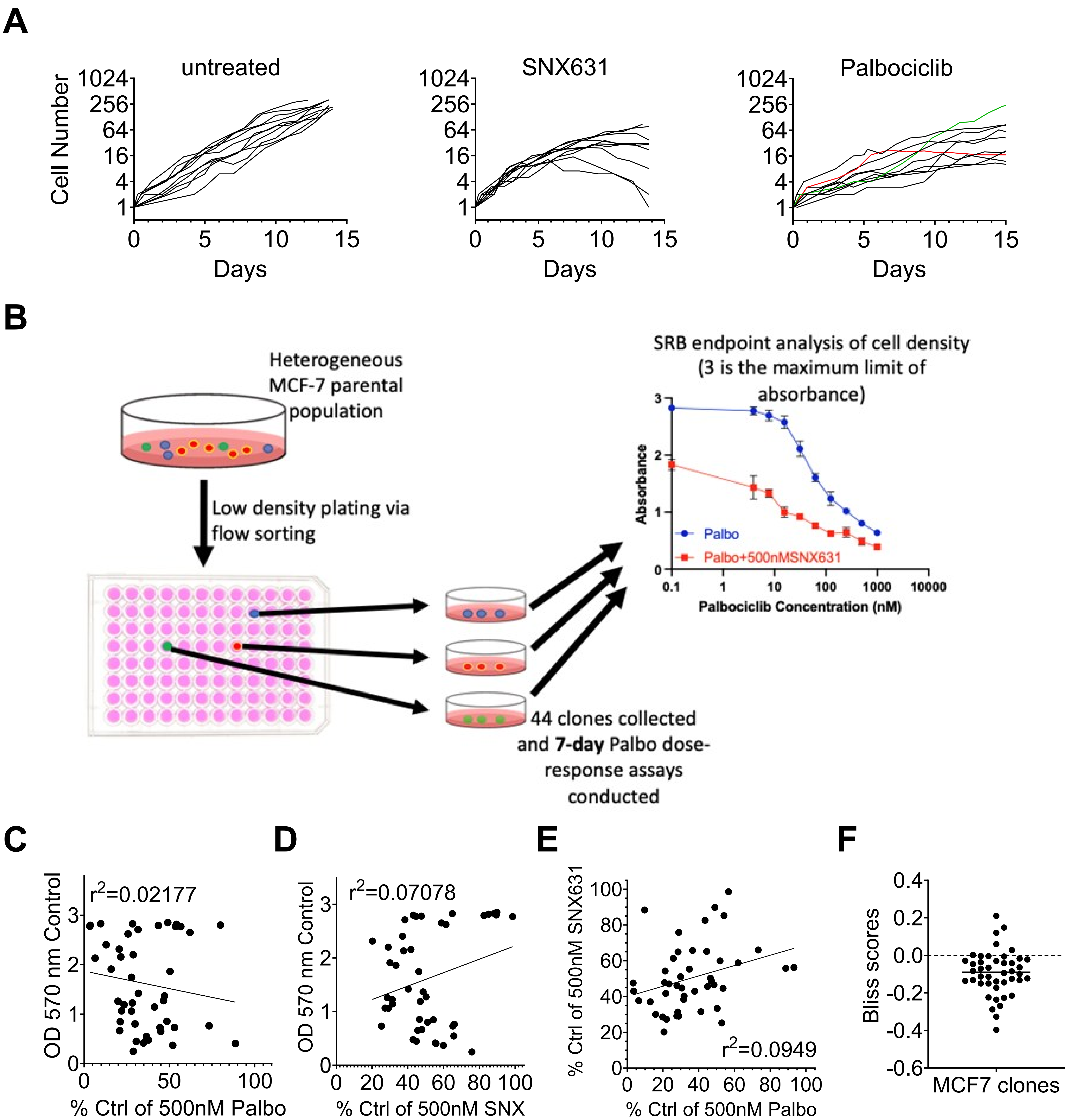

Figure S2. Clonal heterogeneity in MCF-7 cell responses to palbociclib and CDK8/19 inhibition. Related to Figures 2.

(A) Real-time video analysis of 10 clusters of untreated cells (left), cells treated with 500 nM SNX631 (middle), or 500 nM palbociclib (right). Each line represents a cluster originating from a single cell, with green and red lines highlighting clusters of interest under palbociclib treatment. **(B)** Diagram of subcloning of MCF-7 cells; 44 subpopulations were generated using low-density plating. **(C–D)** Correlation curves visualizing characteristics of MCF-7 clones following a 7-day SRB viability assay. Correlation coefficients were determined by linear regression analysis in GraphPad Prism 10. **(E)** Correlation of the effects of 500 nM palbociclib and 500 nM SNX631 on 44 MCF-7 clones (7-day cell growth assay). **(F)** Synergy analysis of 44 MCF-7 clones showing Bliss scores calculated from the effects of 500 nM palbociclib and 500 nM SNX631. Data are represented as mean +/- SEM.

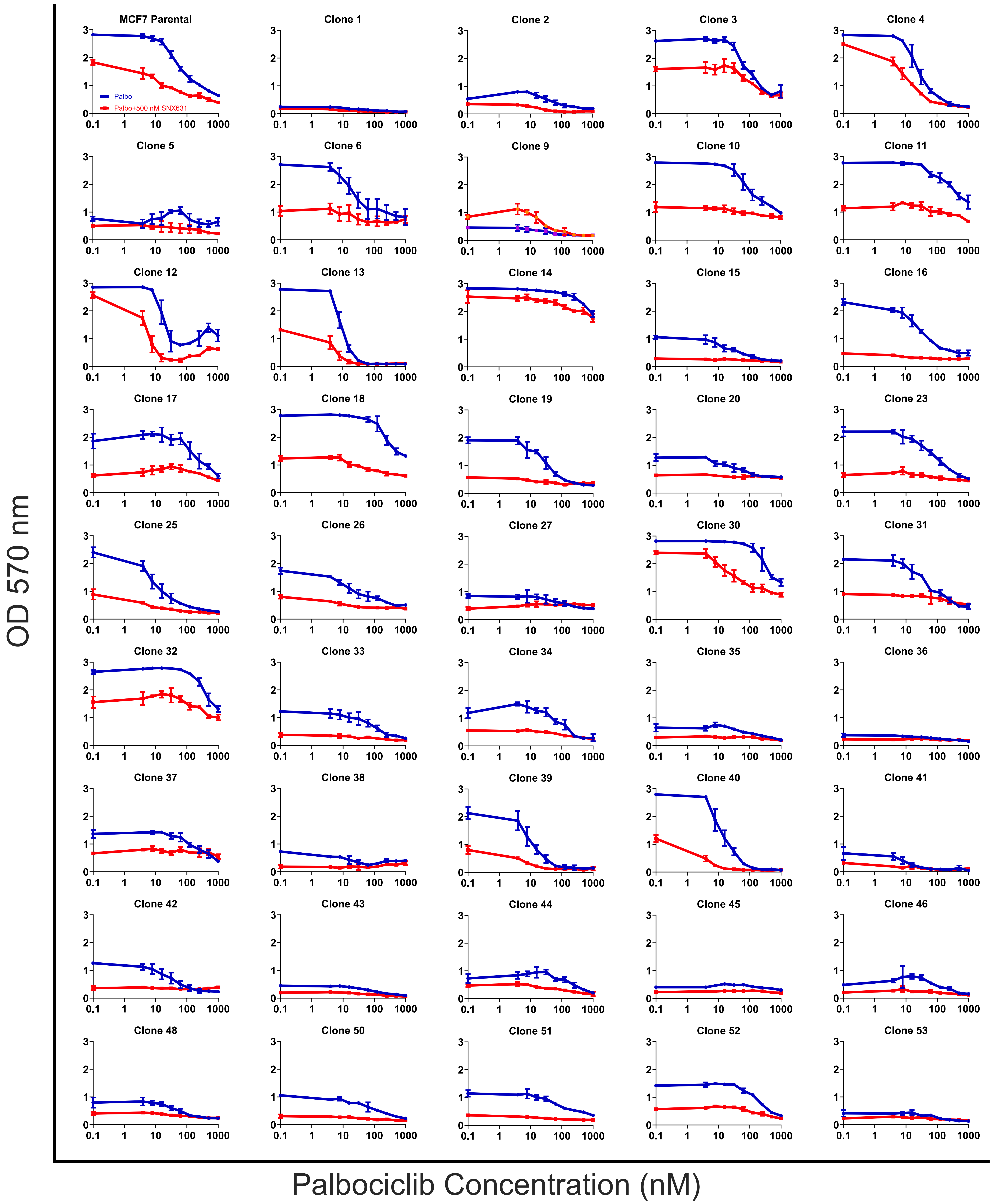

Figure S3. Drug response profiling of single-cell MCF-7 clones to palbociclib and CDK8/19 inhibition. Related to Figures 2.

Single-cell clones were isolated and tested for their sensitivity to palbociclib, SNX631, and the combination, and compared to their parental population (top left). Parameters of the dose–response curves were determined using the IC50 of the parental population. Data are represented as mean +/- SEM.

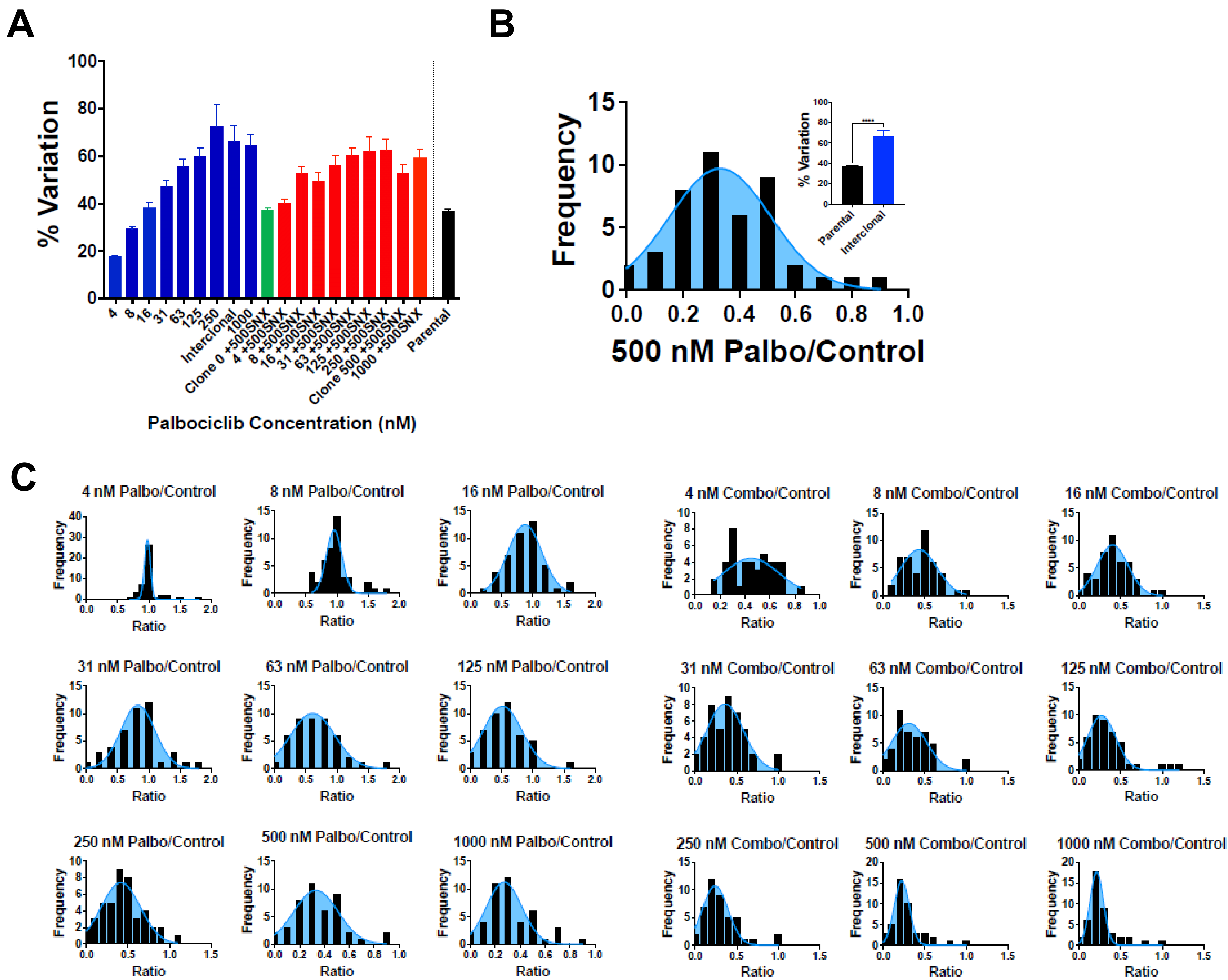

Figure S4. Adaptation to palbociclib occurs regardless of transient sensitivity. Related to Figures 2.

(A) A Percent coefficient of variation between the 44 clones' response to each palbociclib and combination treatment group compared to variation between 10 biological repeats of MCF-7 parental cell line to 500 nM palbociclib. **(B)** Histogram displaying the distribution of relative drug sensitivity to 500 nM palbociclib across 44 MCF-7 subclones. A Gaussian distribution was fit to the data using GraphPad Prism's nonlinear regression function. **(C)** Histograms displaying the distribution of relative drug sensitivity at various concentrations of palbociclib and SNX631+palbociclib combination. A Gaussian distribution was fit to the data using GraphPad Prism's nonlinear regression function. Data are represented as mean +/- SEM.

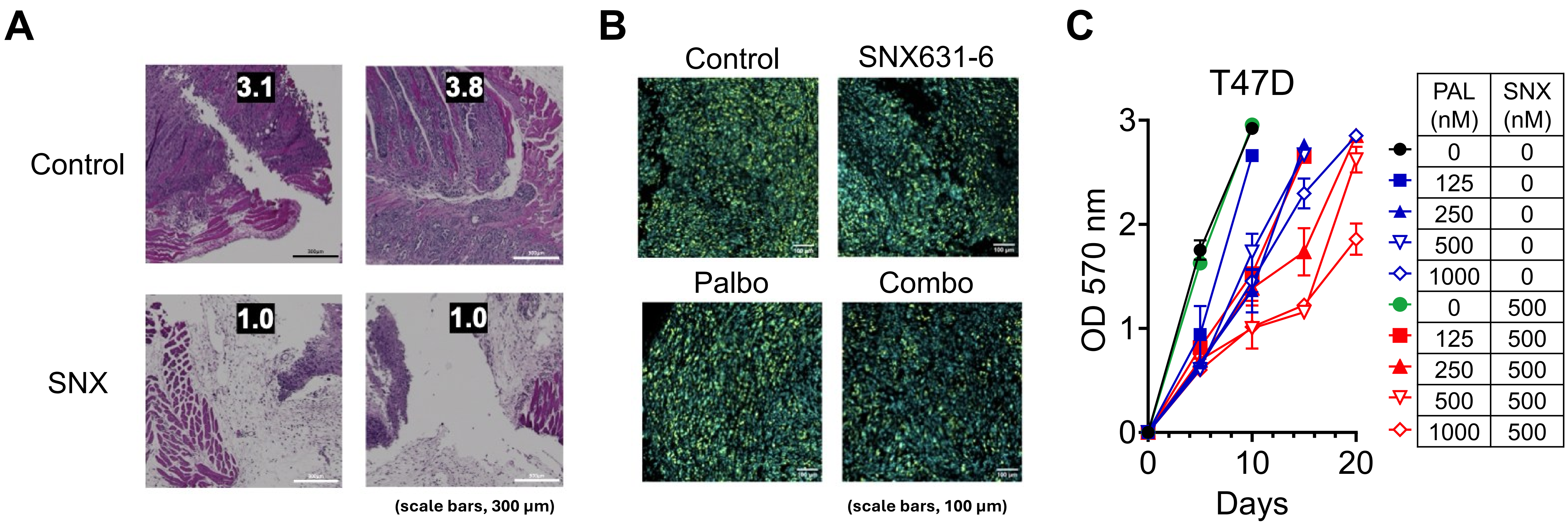

PAL (nM)	SNX (nM)
0	0
125	0
250	0
500	0
1000	0
0	500
125	500
250	500
500	500
1000	500

Figure S5. Effects of CDK8/19 inhibition on MCF-7 tumor invasion in vivo and on palbociclib responses in T47D cell proliferation *in vitro*. Related to Figures 3.

(A) Histological sections of dissected tumors stained with hematoxylin and eosin showing representative images and corresponding invasion scores (top left of each image) for MCF-7 tumors invading abdominal muscle in control (top) versus SNX631-6-treated (bottom) mice. Invasion scores, ranging from 0 (no invasion) to 5 (extensive invasion), were assigned based on the depth of tumor infiltration into muscle tissue (n = 10 tumors per treatment group). **(B)** Representative composite images of Ki67 (yellow)-labeled sections counterstained with DAPI (blue). **(C)** Twenty-day Sulforhodamine B (SRB) viability analyses performed on T47D cells treated with concentrations greater than their IC50 of palbociclib with (red lines) and without (blue lines) 500 nM SNX631. Data are represented as mean +/- SEM.

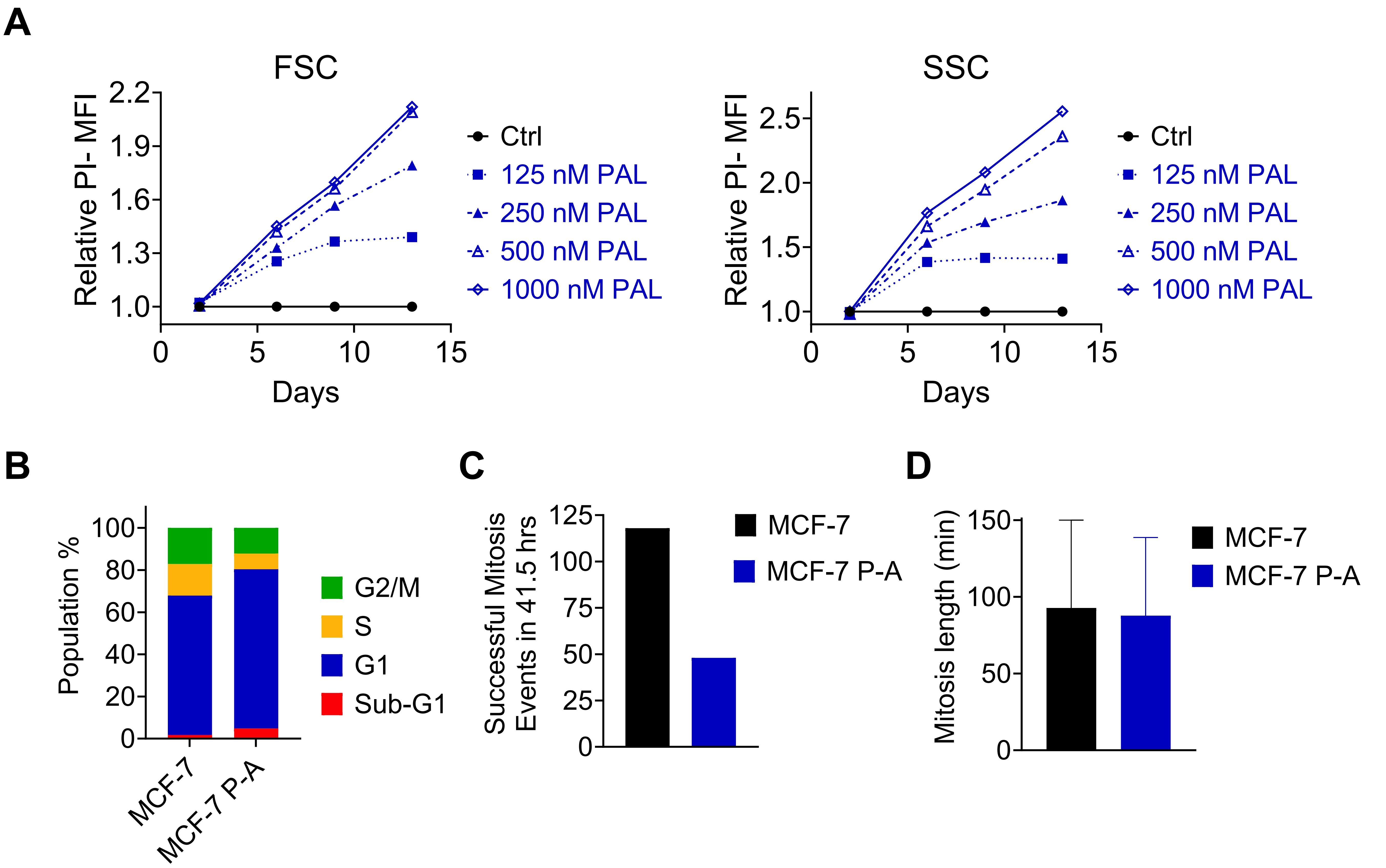

Figure S6. Effects of long-term palbociclib treatment on cell size, cell-cycle distribution, and mitotic dynamics in MCF-7 cells. Related to Figures 4.

(A) Forward scatter (FSC; left) and side scatter (SSC; right) quantification of palbociclib's effect on cell size and internal complexity over time by flow cytometric analysis, measured as mean fluorescence intensity (MFI). **(B)** Cell-cycle profile of MCF-7 cells treated with 0.1% DMSO vehicle control or 500 nM palbociclib for 42 days. **(C–D)** Number (**C**) and duration (**D**) of mitosis events quantified by a 41.5-hour microscope image time course of control and 42-day palbociclib-adapted (P-A) groups.

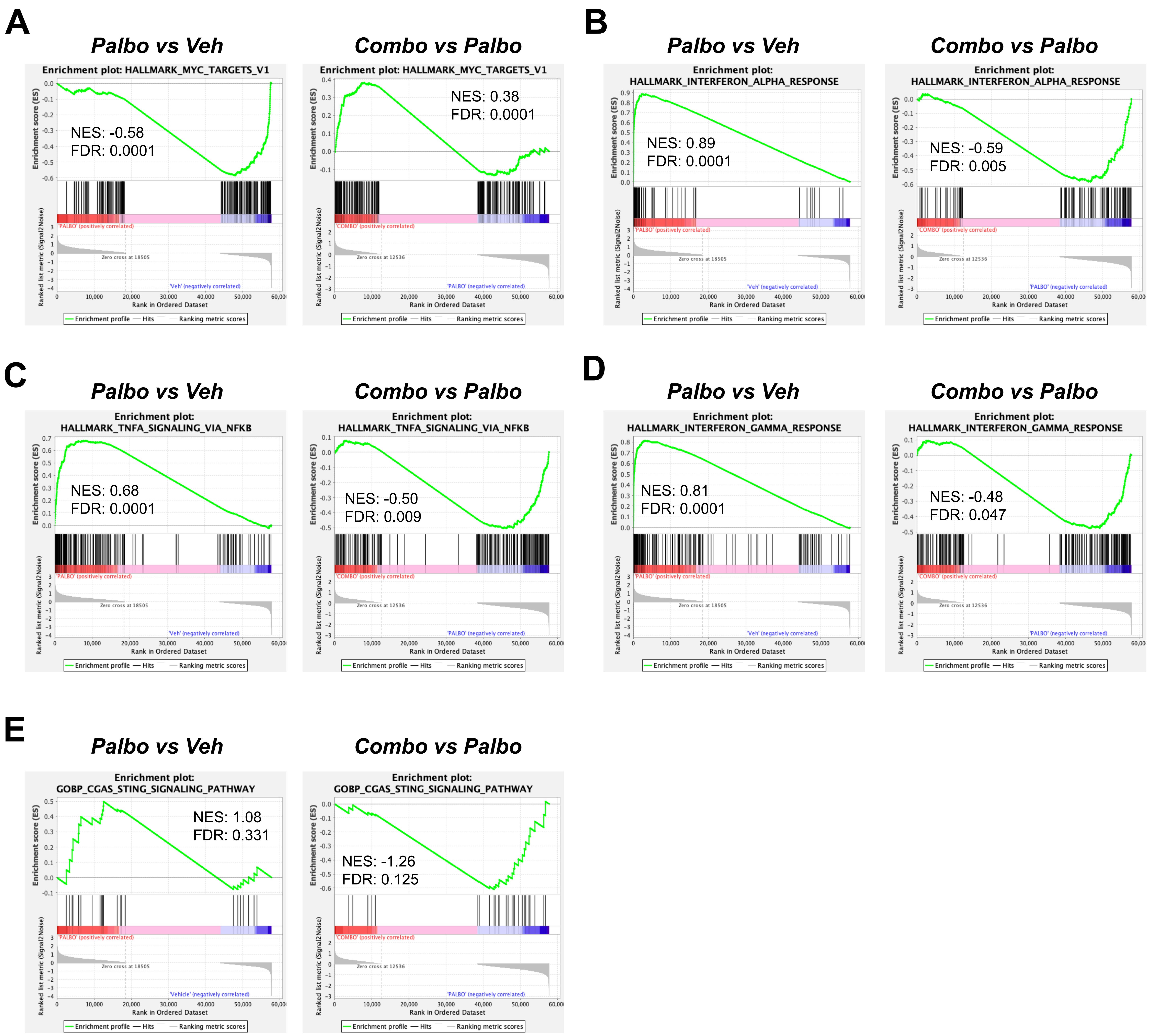

Figure S7. Gene set enrichment analysis (GSEA) of signaling pathways in MCF-7 tumors treated with palbociclib or combination therapy *in vivo*. Related to Figures 6.

(A–E) Enrichment score plots of GSEA Hallmark gene sets for MYC Targets V1 (**A**), Interferon-alpha (**B**), Interferon-gamma (**C**), TNF-alpha (**D**), and cGAS–STING (**E**) pathways comparing *in vivo* palbociclib vs vehicle (left) and *in vivo* combination vs palbociclib (right).

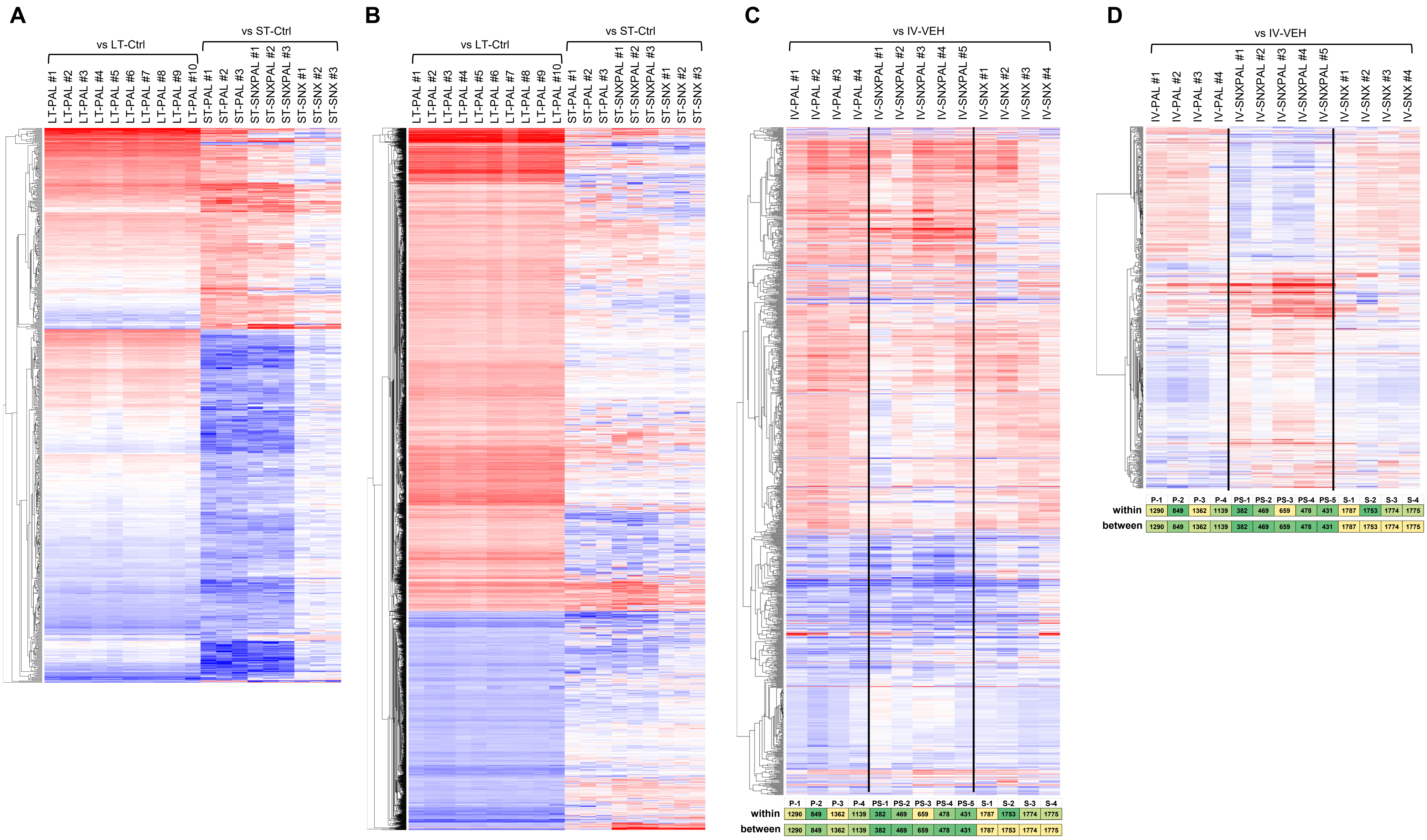

Figure S8. Transcriptomic profiling of palbociclib treatment and its combination with CDK8/19i *in vitro* and *in vivo*. Related to Figure 6

(A–D) Heatmaps displaying individual replicate fold change relative to corresponding control samples with unsupervised hierarchical clustering of 711 DEGs for ST-PAL vs ST-DMSO (**A**), 3,599 DEGs for LT-PAL vs LT-DMSO (**B**), 1,023 DEGs for IV-Palbo vs IV-Vehicle (**C**), and 515 DEGs for IV-Combo vs IV-Palbo (**D**). IV plots include terminal tumor sizes listed above each corresponding replicate and conditionally formatted to label the largest (yellow) to smallest (green) tumors within (top) and between (bottom) treatment groups. DEGs were defined by a fold change >1.5 and an FDR <0.05. Heatmaps were generated using Morpheus (https://software.broadinstitute.org/morpheus).

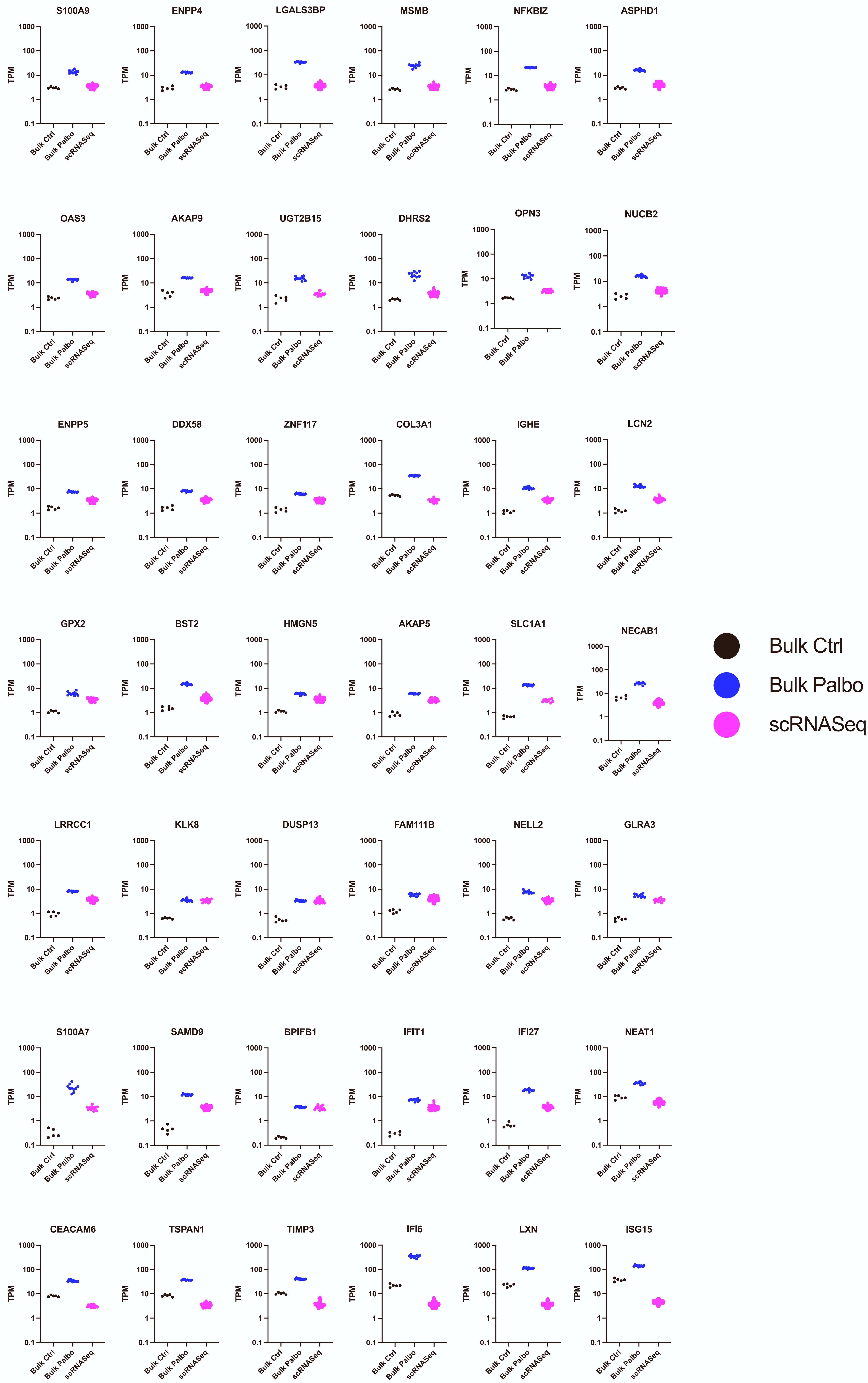
S100A9
ENPP4
LGALS3BP
MSMB
NFKBIZ
ASPHD1
OAS3
AKAP9
UGT2B15
DHRS2
OPN3
NUCB2
ENPP5
DDX58
ZNF117
COL3A1
IGHE
LCN2
GPX2
BST2
HMGN5
AKAP5
SLC1A1
NECAB1
LRRCC1
KLK8
DUSP13
FAM111B
NELL2
GLRA3
S100A7
SAMD9
BPIFB1
IFIT1
IFI27
NEAT1
CEACAM6
TSPAN1
TIMP3
IFI6
LXN
ISG15
TPM
1000
100
10
1
0.1
Bulk Ctrl
Bulk Palbo
scRNASeq

Figure S9. Single-cell expression patterns of long-term palbociclib–associated DEGs in MCF-7 cells. Related to Figure 6

(A) Dot plots of 42 DEGs identified in LT-PAL (log2(FC) $\geq$ 2; FPKM > 5) with nonzero expression in 977 single MCF-7 cells, generated using Prism 10. Plots are ordered from top left to bottom right according to the similarity of single-cell TPM expression to bulk LT-DMSO RNA-seq profiles.
